# Molecular and metabolic mechanisms of quercetin in liver fibrosis: an integrated network pharmacology, metabolomics, and molecular dynamics study

**DOI:** 10.3389/fphar.2026.1838052

**Published:** 2026-08-27

**Authors:** Xiaowen Song, Tianfu Guo, Yaxin Li, Jiali Liu, Shufang Niu, Donghua Zheng, Hongxun Xue, Jianfang Sun, Feng Bai, Zhenwang Wang, Wanfu Bai

**Affiliations:** 1 Department of Pharmacy, Baotou Medical College, Baotou, China; 2 The First Affiliated Hospital of Baotou Medical College, Baotou, China; 3 Institute of Bioactive Substance and Function of Mongolian Medicine and Chinese Materia Medica, Baotou Medical College, Baotou, China

**Keywords:** liver fibrosis, metabolomics, molecular docking, molecular dynamics simulation, network pharmacology, quercetin

## Abstract

**Introduction:**

Liver fibrosis, a severe chronic liver injury sequela, lacks effective therapies. Quercetin, a natural flavonoid, exhibits multi-target anti-fibrotic potential.

**Methods:**

This study integrated network pharmacology,metabolomics, molecular docking, and molecular dynamics simulations to investigate its mechanisms.

**Results:**

Network analysis identified 203 overlapping targets, with core targets including protein kinase B (AKT1), interleukin-6 (IL6), tumor protein p53 (TP53), and tumor necrosis factor (TNF), which were enriched in PI3K-Akt and TNF signaling pathways. In CCl4- induced rats (quercetin 50 mg/kg/day), quercetin alleviated pathological damage, significantly reduced collagen area by 43.1% (P = 0.006), and lowered serum alanine aminotransferase, aspartate aminotransferase, and type III procollagen levels. Metabolomics revealed 32 differential metabolites, implicating multiple metabolic pathways including lipid and amino acid metabolism. Molecular docking showed strong binding to AKT1 (5.8 kcal/mol) and TNF (7.2 kcal/ mol), with stable complexes over 100 ns simulations. QPCR was used to detect the expression of PI3K/AKT1 core targets in liver tissue, and experimental verification confirmed the predicted key targets.

**Conclusion:**

This multi-faceted study elucidates quercetin’s anti-fibrotic mechanism, laying a foundation for its therapeutic development.

## Graphical Abstract

**Figure:**
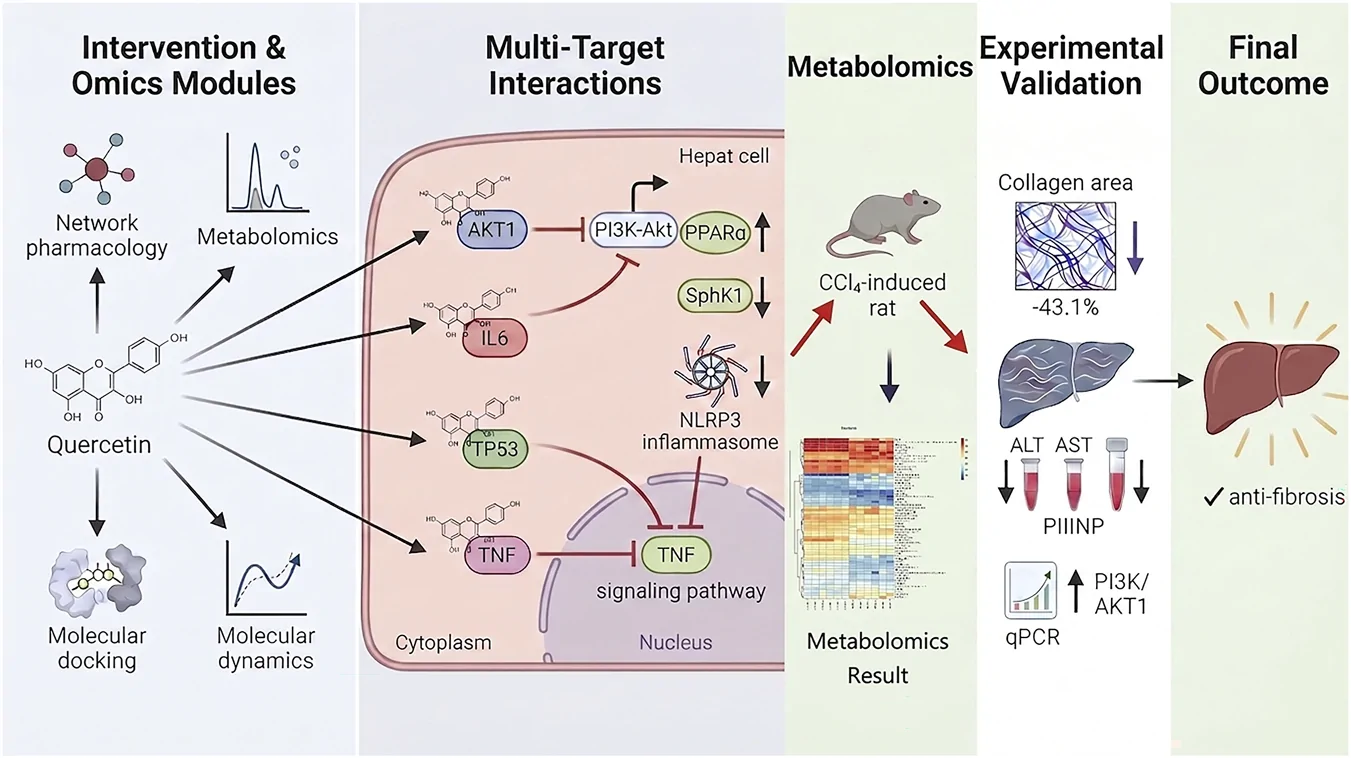

## 1 Introduction

Liver fibrosis, a pathological consequence of chronic liver injury, is characterized by excessive deposition of extracellular matrix (ECM), which disrupts liver architecture and can progress to cirrhosis. The activation of hepatic stellate cells (HSCs) is a central event in this process (Roehlen et al., 2020; Parola and Pinzani, 2019).

Current therapies primarily target disease etiology, leaving a critical need for effective anti-fibrotic agents (Hernández-Gea and Friedman, 2011). Quercetin, a natural flavonoid with known antioxidant and anti-inflammatory properties, shows therapeutic potential (Kashyap et al., 2019; Banerjee et al., 2025). However, its comprehensive, multi-target mechanism against liver fibrosis remains to be fully elucidated.

Systems biology approaches like network pharmacology and metabolomics, combined with computational validation, offer a powerful strategy to address this gap (Guo et al., 2022; Li X. et al., 2023). Indeed, network pharmacology has been increasingly applied to investigate the mechanisms of natural products in various liver diseases, including non-alcoholic fatty liver disease and alcoholic liver disease (Ren et al., 2024; Yao et al., 2024). However, a truly comprehensive understanding requires the integration of predictive, experimental, and simulational evidence into a cohesive framework.

Although previous studies have demonstrated that quercetin inhibits hepatic stellate cell activation and reduces collagen deposition primarily via antioxidant effects or the TGF-β1 signaling pathway (Kashyap et al., 2019; Banerjee et al., 2025), most of these investigations examined a restricted set of targets and did not address systemic metabolic reprogramming. In contrast, our study establishes a systems-level mechanistic framework by integrating network pharmacology, metabolomics, and molecular dynamics simulations. Notably, we attribute quercetin’s anti-fibrotic action to the reversal of linoleic acid metabolism, glycerophospholipid metabolism, and GPI-anchor biosynthesis—a finding that, to our knowledge, remains unreported. This study therefore aimed to employ a multi-level strategy—from network-based target prediction and *in vivo* validation to metabolite profiling and atomic-level simulation—to systematically uncover the molecular and metabolic mechanisms underlying quercetin’s anti-fibrotic effects. This integrative approach provides a closed loop of evidence, from systemic hypothesis generation to mechanistic confirmation, offering a robust model for elucidating the polypharmacology of natural products.

## 2 Materials and methods

### 2.1 Network pharmacology analysis

The structure of quercetin (PubChem CID: 5280343) was retrieved from PubChem, and its drug-likeness (DL ≥ 0.18) and oral bioavailability (OB ≥ 30%) were evaluated. Putative targets of quercetin were collected from TCMSP (OB ≥ 30%, DL ≥ 0.18), BATMAN-TCM (score >0.84), and SwissTargetPrediction (probability >0), restricted to *Homo sapiens* (Wishart, 2019). Only targets with a SwissTargetPrediction probability >0.8 were retained, then standardized using UniProt and deduplicated.

Liver fibrosis-associated genes were sourced from GeneCards, OMIM, and DisGeNET using “liver fibrosis” as the search term, with a relevance score threshold ≥10. Genes were consolidated and deduplicated. For PPI network construction via STRING, a minimum interaction confidence threshold of 0.4 was used.

The intersection between quercetin targets and liver fibrosis genes was identified using the VennDiagram package in R. Potential therapeutic targets were further filtered (|logFC| ≥ 1.5, adjusted *P*-value <0.05) and visualized.

The overlapping targets were imported into the STRING database (species: *H. sapiens*, confidence >0.4) to construct a Protein-Protein Interaction (PPI) network, which was visualized in Cytoscape 3.8.2.

Network topology was analyzed using the CytoNCA plugin. Key targets were identified by filtering nodes with Degree Centrality ≥ 2 × median, Betweenness Centrality ≥0.05, and Closeness Centrality ≥0.519.

A “quercetin-target-liver fibrosis” network was constructed in Cytoscape to visualize regulatory relationships, with node size scaled by Degree Centrality.

The CytoHubba plugin (MCC algorithm) ranked nodes in the PPI network, and the top 30 targets were selected as core targets. Functional modules within the PPI network were identified using the MCODE plugin (parameters: degree cutoff = 2, node score cutoff = 0.2, k-core = 2, max depth = 100). The top three modules were analyzed.

Gene Ontology (GO) and Kyoto Encyclopedia of Genes and Genomes (KEGG) pathway enrichment analyses were performed using the clusterProfiler package in R (*P*-value and q-value <0.05) (Zhao et al., 2022). The top 10 enriched terms from each GO category (Biological Process, Cellular Component, Molecular Function) were visualized.

### 2.2 Animal experimental study

Eighteen male specific pathogen-free (SPF) Sprague-Dawley rats (6–8 weeks, 180–220 g) were housed under controlled conditions. All procedures were approved by the Animal Ethics Committee of Baotou Medical College (IACUC-2020-038).

Quercetin (purity ≥98%, catalog No. SQ8030) and silymarin (purity ≥98%, catalog No. IS3400) were purchased from Beijing Solarbio Technology Co. Ltd, Beijing, China. Carbon tetrachloride (CCl_4_, catalog No. 319961) and corn oil (catalog No. C8267) were obtained from Sigma-Aldrich, St. Louis, MO, United States. Enzyme-linked immunosorbent assay (ELISA) kits for alanine aminotransferase (ALT), aspartate aminotransferase (AST), collagen type IV (COLIV), type III procollagen (PC-III), hyaluronic acid (HA), laminin (LN), interleukin-1β (IL-1β), and tumor necrosis factor-α (TNF-α) were purchased from Nanjing Jiancheng Bioengineering Institute (Nanjing, China; catalog Nos. C009-2-1 for ALT, C010-2-1 for AST, H165-1-2 for COLIV, H212-1-1 for PC-III, H188-1-2 for HA, H121-1-2 for LN, H002-1-2 for IL-1β, and H052-1-2 for TNF-α). Quercetin was suspended in 1% sodium carboxymethyl cellulose (CMC-Na, catalog No. C5678, from Sigma-Aldrich, St. Louis, MO, United States) for daily administration.

Silymarin, a standardized flavonolignan extract from milk thistle (Silybum marianum), is a well-established hepatoprotective agent with proven anti-fibrotic activity. It inhibits hepatic stellate cell (HSC) activation, reduces collagen deposition, and suppresses oxidative stress and inflammatory signaling (e.g., NF-κB and TGF-β1 pathways). Therefore, silymarin was selected as the positive control in this study to validate the CCl_4_-induced liver fibrosis model and to benchmark the anti-fibrotic efficacy of quercetin.

Rats were randomly divided into four groups (*n* = 6): Control (CON), Model (MOD), Quercetin (QR, 50 mg/kg/day), and Silymarin (SM, 100 mg/kg/day). The dose of silymarin (100 mg/kg/day) was chosen based on previous reports of its protective effects in rodent liver fibrosis models, including studies in which 100 mg/kg silymarin showed significant protective effects in reducing serum ALT/AST and attenuating collagen deposition in CCl_4_-treated rats and mice, ensuring a reliable positive control for comparison with quercetin treatment (Wu, 2017; Cao et al., 2009). Liver fibrosis was induced in MOD, QR, and SM groups by intraperitoneal injection of 40% CCl_4_ in corn oil (2 mL/kg, twice weekly) for 8 weeks. CON received equivalent volumes of corn oil and saline. Body weight and health were monitored.

After 8 weeks, blood was collected for serum separation. Liver tissue was harvested: part fixed in formalin for histology, part frozen at −80 °C for analysis.

Fixed liver tissues were paraffin-embedded, sectioned, and stained with Hematoxylin and Eosin (H&E) and Masson’s trichrome. Histopathological scoring for hepatic inflammation and collagen deposition was performed by a blinded pathologist according to the 0–4 grading system adapted from Ruwart et al. (1989) (Ruwart et al., 1989). The criteria were defined as follows: 0 = none; 1 = slight (focal or periportal inflammatory infiltration and minimal collagen deposition); 2 = mild (obvious but localized changes, incomplete septa); 3 = moderate (complete thin septa with bridging fibrosis); 4 = remarkable (thick septa, pseudolobule formation, extensive collagen deposition). Both H&E and Masson’s trichrome-stained sections were evaluated.

For quantitative analysis of collagen deposition, five non-overlapping fields were randomly captured from each Masson-stained section under a light microscope at ×200 magnification. The area of collagen fibers (stained blue) was quantified using ImageJ software (v1.53t, National Institutes of Health, United States) by a blinded observer. The percentage of collagen area was calculated as the ratio of blue-stained area to total tissue area in each field. The mean value from the five fields was taken as the representative value for each animal.

Serum alanine aminotransferase (ALT) and aspartate aminotransferase (AST) activities were determined using commercially available colorimetric assay kits following the manufacturers’ protocols. Absorbance readings were performed at appropriate wavelengths using a microplate reader (Thermo Fisher Multiskan FC, Waltham, MA, United States). For enzyme-linked immunosorbent assay (ELISA) measurements, approximately 100 mg of liver tissue was homogenized in 1 mL ice-cold phosphate-buffered saline (PBS, pH 7.4) containing a protease inhibitor cocktail. The homogenate was centrifuged at 12,000 × g for 15 min at 4 °C, and the supernatant was collected. Total protein concentration in the supernatant was determined using a bicinchoninic acid (BCA) assay kit. Concentrations of collagen type IV (COLIV), procollagen type III (PC-III), hyaluronic acid (HA), laminin (LN), interleukin-1β (IL-1β), and tumor necrosis factor-α (TNF-α) were quantified using specific rat ELISA kits. Each sample was analyzed in duplicate, and the optical density was recorded at 450 nm using the same microplate reader. All measured values were normalized to total protein content and expressed as appropriate units (ng/mg protein for COLIV, PC-III, LN; pg/mg protein for IL-1β, TNF-α; μg/g liver tissue for HA). Standard curves were included in each plate to ensure data quality. All procedures strictly followed the kit instructions (Wang et al., 2017).

For metabolomics, serum proteins were precipitated with cold methanol containing an internal standard. Metabolites were analyzed by ultra-performance liquid chromatography quadrupole time-of-flight mass spectrometry (UPLC-QTOF-MS) (Hou et al., 2023). Data were processed, and an OPLS-DA model was constructed. Differential metabolites were initially screened using criteria of VIP >1.0 and *P* < 0.05 from the OPLS-DA model. To control the false discovery rate (FDR), the Benjamini–Hochberg method was then applied, and only metabolites with an adjusted *P*-value <0.05 were considered statistically significant. The identified metabolites were annotated using HMDB and KEGG.

To monitor instrument stability and ensure data reproducibility throughout the metabolomics analysis, pooled quality control (QC) samples were prepared by mixing equal volumes of all individual biological samples, thereby creating a representative QC sample reflecting the average metabolic profile of the entire sample cohort. The pooled QC samples were interspersed into the analytical sequence every 10–15 experimental samples. In addition, 3–5 consecutive QC injections were performed at the beginning and at the end of each analytical run to equilibrate the mass spectrometry system and to monitor baseline drift. All QC samples were analyzed under the same chromatographic and mass spectrometric conditions as the experimental samples. Key system performance metrics, including retention time stability, peak area repeatability, and mass spectrometric response intensity, were systematically monitored to assess the reliability of the analytical system. This QC strategy adheres strictly to the established guidelines for untargeted metabolomics proposed by Broadhurst et al. (2018).

To assess the reliability of the multivariate statistical models and to mitigate the risk of overfitting, internal cross-validation was performed on the established PLS-DA and OPLS-DA models. The entire dataset was randomly partitioned into k subsets. In each iteration, k−1 subsets served as the training set for model construction, while the remaining subset functioned as the test set for evaluating predictive performance. Two core parameters were calculated based on the cross-validation results: the coefficient of determination (R^2^Y) and the predictive ability coefficient (Q^2^). R^2^Y reflects the explanatory power of the model for the Y-variables (group classification), while Q^2^ represents the predictive generalizability of the model. R^2^Y and Q^2^ values approaching 1 indicate a good model fit and strong predictive ability, thus supporting the validity and reliability of the classification model.

Metabolite annotation and identification were performed by matching the acquired experimental data against public databases and in-house standard databases. For each metabolite feature, the accurate mass, retention time, and fragmentation pattern were rigorously matched with those of reference substances validated using isotope-labeled internal standards or chemical standards. This identification strategy aligns with the highest confidence level for metabolite annotation in metabolomics studies. The rigorously validated metabolite identification results are reliable for targeted quantitative analysis and provide a basis for subsequent verification of key differential metabolites in future studies.

Statistical analysis was conducted with SPSS. Data are presented as mean ± SD. Comparisons among multiple groups were performed using one-way ANOVA followed by Tukey’s honest significant difference (HSD) *post hoc* test. For comparisons between two groups, Student's t-test was employed. *P*-values <0.05 were considered statistically significant.

### 2.3 Quantitative real-time PCR (qPCR)

To validate the network pharmacology predictions, quantitative real-time PCR (qPCR) was performed on liver tissue samples. Based on the network pharmacology and KEGG enrichment analyses, five core molecules in the PI3K-Akt signaling axis (*AKT1*, *PIK3CA*, *PTEN*, *MTOR*, and *GSK3B*) were selected for validation. The predicted expression trends of these genes across the three experimental groups (CON, MOD, QR) are as follows: *AKT1* (↑ in MOD vs. CON, ↓ in QR vs. MOD), *PIK3CA* (↑ in MOD vs. CON, ↓ in QR vs. MOD), *PTEN* (↓ in MOD vs. CON, ↑ in QR vs. MOD), *MTOR* (↑ in MOD vs. CON, ↓ in QR vs. MOD), *GSK3B* (↓ in MOD vs. CON, ↑ in QR vs. MOD). Total RNA was extracted from approximately 50–100 mg of liver tissue from each group (CON, MOD, QR, SM; *n* = 6 per group) using TRIzol reagent (Invitrogen, Carlsbad, CA, United States). RNA concentration and purity were determined using a NanoDrop ND-2000 spectrophotometer (Thermo Fisher Scientific, Waltham, MA, United States). First-strand cDNA was synthesized from 1 μg of total RNA using a PrimeScript RT reagent kit with gDNA Eraser (Takara Bio Inc., Shiga, Japan). qPCR was performed using TB Green Premix Ex Taq II (Takara) on a LightCycler 480 II Real-Time PCR System (Roche Diagnostics, Basel, Switzerland). The thermal cycling protocol was: 95 °C for 30 s, followed by 40 cycles of 95 °C for 5 s and 60 °C for 30 s. A melting curve analysis was performed after each run to confirm amplification specificity. The primer sequences are listed in Table 1. GAPDH was used as the internal control. Relative expression levels were calculated using the 2^(−ΔΔCt) method. All reactions were performed in triplicate.

**TABLE 1:** Primer sequences used for qPCR.

Gene	Forward primer (5′→3′)	Reverse primer (5′→3′)
*AKT1*	TGT​CAT​CGA​ACG​CAC​CTT​CC	ACA​CCT​CCA​TCT​CTT​CAG​CC
*PIK3CA*	TTA​TTG​AAC​CAG​TAG​GCA​ACC​G	GCT​ATG​AGG​CGA​GTT​GAG​ATC​C
*PTEN*	CAA​TGT​TCA​GTG​GCG​AAC​TT	GGC​AAT​GGC​TGA​GGG​AAC​T
*MTOR*	ACC​GGC​ACA​CAT​TTG​AAG​AAG	CTC​GTT​GAG​GAT​CAG​CAA​GG
*GSK3B*	TGG​CAG​CAA​GGT​AAC​CAC​AG	CGG​TTC​TTA​AAT​CGC​TTG​TCC​TG
*GAPDH*	CTG​GAG​AAA​CCT​GCC​AAG​TAT​G	GGT​GGA​AGA​ATG​GGA​GTT​GCT

### 2.4 Molecular docking

The three-dimensional (3D) structure of quercetin (PubChem CID: 5280343) was retrieved from the PubChem database. The previously resolved crystal structures of AKT1 (PDB ID: 1H10) and TNF (PDB ID: 2AZ5) were downloaded from the RCSB Protein Data Bank. Protein structures were prepared by removing water molecules and co-crystallized ligands, adding polar hydrogens, and assigning Gasteiger charges using AutoDockTools. Quercetin was prepared by detecting rotatable bonds. A grid box was defined to cover the putative ligand-binding pocket of each protein. Docking simulations were performed using AutoDock Vinawith standard parameters (exhaustiveness = 8 or 32, depending on the protein) (Deng et al., 2025). The binding pose with the lowest binding energy (kcal/mol) was selected for each complex. Binding energy < −5.0 kcal/mol was considered indicative of potential binding, and < −7.0 kcal/mol as strong binding. Visualization of interactions, including hydrogen bonds, hydrophobic contacts, and van der Waals forces, was carried out using PyMOL and LigPlot+. The docking protocol followed previously described standards.

### 2.5 Molecular dynamics simulation

Molecular dynamics (MD) simulations were performed using GROMACS 2022. The AMBER14SB force field was applied for the proteins, and GAFF2 with AM1-BCC charges was used for quercetin. Each protein-ligand complex was solvated in a cubic periodic box with a minimum distance of 1.0 nm from the protein surface to the box edge using TIP3P water molecules. The system was neutralized by adding counter-ions (Na^+^ or Cl^−^), and additional ions were added to reach a physiological salt concentration (0.15 M). Energy minimization was performed using the steepest descent algorithm until convergence. The system was then equilibrated under NVT conditions (100 ps, 310 K, Berendsen thermostat) followed by NPT conditions (100 ps, 1 bar, Parrinello-Rahman barostat). The production MD run was carried out under NPT conditions for 100 ns with a 2-fs time step. Long-range electrostatic interactions were treated using the Particle Mesh Ewald (PME) method, with a cutoff of 1.0 nm for both van der Waals and Coulomb interactions. Bond lengths involving hydrogen atoms were constrained using the LINCS algorithm. Trajectories were saved every 10 ps. Stability and structural properties were assessed by calculating the root-mean-square deviation (RMSD) of the protein backbone, root-mean-square fluctuation (RMSF) per residue, radius of gyration (Rg), solvent-accessible surface area (SASA), and the number of hydrogen bonds between quercetin and the protein, using GROMACS built-in tools. The simulation protocol is consistent with previous computational studies (Li TT. et al., 2023).

## 3 Results

### 3.1 Network pharmacology analysis results

#### 3.1.1 Identification of quercetin targets

The complete chemical structure of quercetin was sourced from the PubChem database (PubChem CID: 5280343; Canonical SMILES: C1=CC(=C(C=C1C2=C(C(=O)C3=C(C=C(C=C3O2)O)O)O)O)O). A comprehensive search was subsequently conducted across various drug target databases, including TCMSP, BATMAN 2.0, and SwissTargetPrediction. Following the integration and elimination of duplicate entries from these database predictions, a total of 339 potential quercetin targets were compiled.

These targets encompassed a broad spectrum of protein classes, such as kinases, receptors, and transcription factors, highlighting the multi-target and multi-pathway properties of quercetin. This provides a foundational basis for exploring its anti-fibrotic mechanisms through network pharmacology.

#### 3.1.2 Liver fibrosis disease gene collection results

Genes associated with liver fibrosis were systematically compiled from multiple disease genomics databases. Specifically, 1862 disease-associated genes were retrieved from the GeneCards database using the query “liver fibrosis” and applying a Relevance score threshold of greater than 10. Additionally, 53 genes pertinent to the genetic mechanisms of liver fibrosis were extracted from the OMIM database. Following the integration of results from both databases, duplicate entries were eliminated using R, culminating in a final set of 1,868 genes associated with liver fibrosis.

#### 3.1.3 Acquisition of overlapping targets for quercetin in liver fibrosis treatment

**FIGURE 1:**
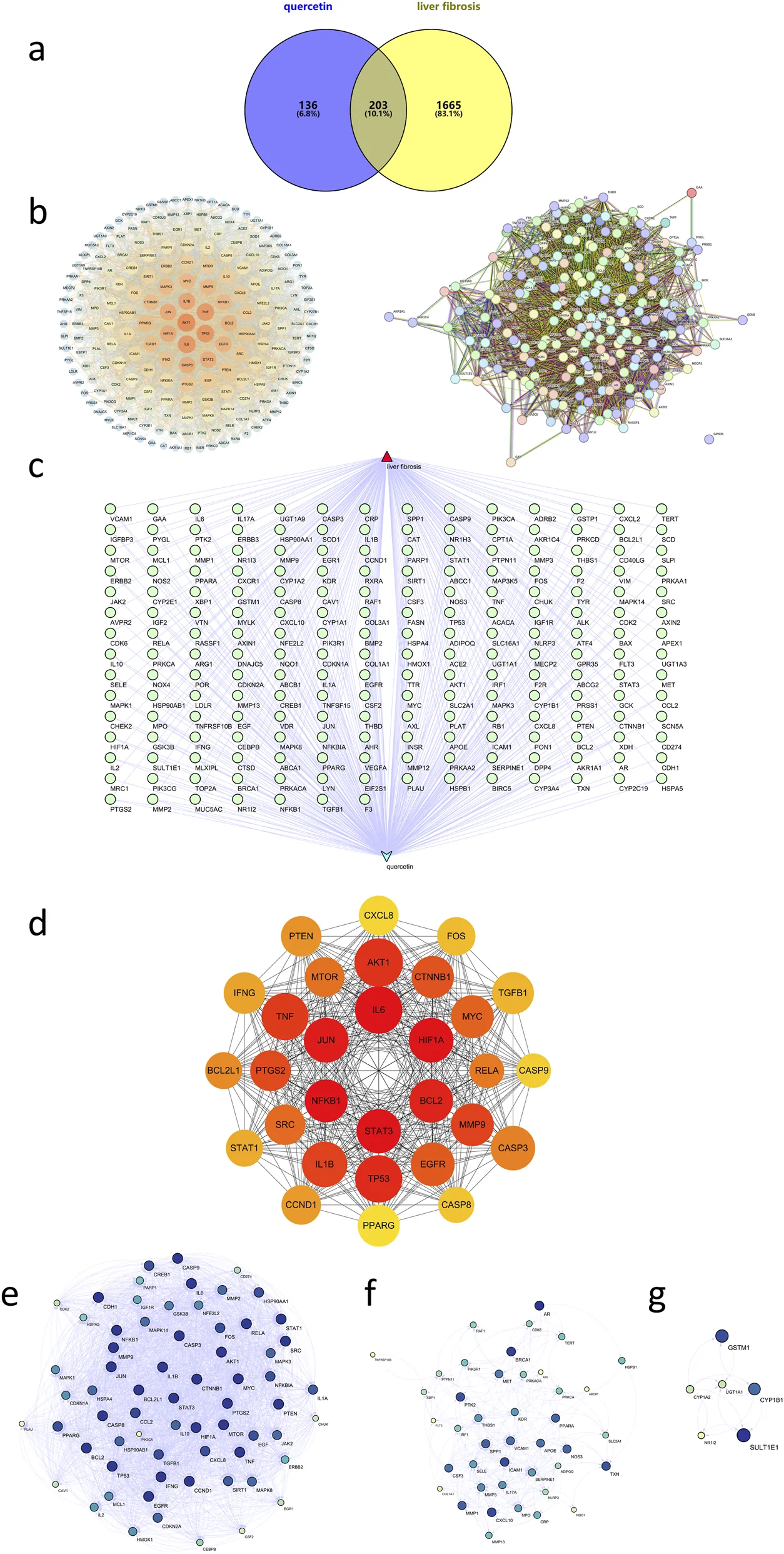
**(a)** Venn diagram showing the intersection between quercetin targets and liver fibrosis-related genes. **(b)** Protein-protein interaction (PPI) network of the overlapping targets. **(c)** The “quercetin-target-liver fibrosis” interaction network. **(d)** The top 30 core targets identified by the Maximal Clique Centrality (MCC) algorithm. **(e–g)** Functional modules (Cluster1, Cluster2, Cluster3) identified within the PPI network by MCODE analysis. Data were generated from network pharmacology analysis with a confidence score threshold >0.4. CON: control; MOD: model; QR: quercetin; SM: silymarin.

The intersection between the 339 potential quercetin targets and the 1,868 liver fibrosis-related genes was examined utilizing the VennDiagram package in R version 4.3.3, which identified 203 overlapping targets (Figure 1a). These overlapping targets represent the direct potential basis for quercetin’s intervention in liver fibrosis. Notably, key genes such as *AKT1, TP53, TNF, IL6,* and *JUN*, which are closely associated with liver fibrosis, were identified among these targets. The Venn diagram provides a visual representation of the overlap between drug targets and disease-related genes, implying that quercetin may exert its anti-liver fibrosis effects through a multi-target synergistic mechanism. The list of overlapping genes was saved in a file named ‘Venn.xlsx’ for subsequent network pharmacology and enrichment analysis.

#### 3.1.4 Protein-protein interaction (PPI) network construction results

The 203 overlapping targets were submitted to the STRING database, with the species specified as “*Homo sapiens*” and a minimum interaction confidence threshold set at 0.4. This analysis resulted in the construction of a protein-protein interaction (PPI) network comprising 202 nodes and 6,341 interaction edges. Through network analysis, it was determined that the constructed network demonstrated a high level of connectivity, with an average node degree of 31.4, suggesting substantial biological associations among the identified targets.

When ranked by node degree, the top ten targets were identified as *AKT1* (degree = 165), *IL6* (degree = 159), *TP53* (degree = 158), *TNF* (degree = 157), *IL1B* (degree = 148), *JUN* (degree = 143), *HIF1A* (degree = 143), *CASP3* (degree = 142), *STAT3* (degree = 141), and *EGFR* (degree = 138). These highly interactive targets are likely to serve as core regulatory molecules in the anti-fibrotic mechanism of quercetin (see Figure 1b).

#### 3.1.5 Compound-target-disease network construction results

Utilizing the 203 overlapping targets, a “quercetin-target-liver fibrosis” tripartite interaction network was developed, comprising 205 nodes, which include quercetin, the 203 targets, and the liver fibrosis node, and encompassing a total of 508 interactions (refer to Figure 1c). Topological analysis of this network revealed an average connectivity of 2.48.

This network provides a visual representation of the potential mechanisms by which quercetin exerts its effects on liver fibrosis through multi-target and multi-pathway synergistic actions. Targets with elevated degree values, including *MMP2*, *XDH*, *MPO*, *CYP1B1*, *ABCG2*, *EGFR*, *INSR*, *PARP1*, *AKT1*, and *MMP9*, are likely to serve as crucial bridging elements within the network, underscoring their central significance in the anti-fibrotic effects of quercetin.

#### 3.1.6 Key target screening results

Key targets within the protein-protein interaction (PPI) network were identified using the CytoHubba plugin in Cytoscape 3.8.2, employing the Maximal Clique Centrality (MCC) algorithm. This algorithm is adept at pinpointing core proteins with substantial topological significance by assessing the frequency of a node’s occurrence across all maximal cliques in the network, making it particularly effective for detecting highly interactive protein clusters. The top 30 core targets ranked by MCC score were identified (Figure 1d), including *NFKB1*, *STAT3*, *IL6*, *HIF1A* (all with a score of 5.94 × 10^52^), *JUN* (5.94 × 10^52^), *BCL2* (5.94 × 10^52^), *TP53* (5.94 × 10^52^), *AKT1* (5.94 × 10^52^), *TNF* (5.94 × 10^52^), and *IL1B*, *MMP9* (both with a score of 5.94 × 10^52^).

The MCC scores of these targets were significantly higher than those of other nodes in the network, indicating their high centrality and potential role as key regulatory hubs in quercetin’s intervention in liver fibrosis.

#### 3.1.7 Protein cluster analysis results

The functional module analysis of the protein-protein interaction (PPI) network was performed utilizing the MCODE plugin in Cytoscape version 3.8.2, with the parameters set as follows: degree cutoff = 2, node score cutoff = 0.2, k-core = 2, and maximum depth = 100. This analysis resulted in the identification of eight modules, with the three highest-scoring modules selected for detailed examination.

Cluster 1, the most significant module with a score of 60.727, comprised 67 genes and 2004 interaction edges. The core genes of this module included *CDH1*, *HIF1A*, *BCL2*, *STAT3*, *IL1B*, *BCL2L1*, *SRC*, *IFNG*, *PTEN*, and *NFKB1*. This module was predominantly associated with biological processes such as the regulation of apoptosis, inflammatory response, and cell proliferation (refer to Figure 1e).

Cluster 2, with a score of 12.732, contained 42 genes and 261 edges, featuring *AR*, *BRCA1*, and *CXCL10* as its core genes. This cluster was potentially linked to mechanisms such as hormone response, DNA damage repair, and immune regulation (see Figure 1f).

Cluster 3, scoring 5.2, consisted of 6 genes and 13 edges. Key genes in this cluster included *SULT1E1*, *GSTM1*, and *CYP1B1*, and it was primarily involved in xenobiotic metabolism and detoxification processes, suggesting that quercetin may exert synergistic effects through metabolic regulation (illustrated in Figure 1g).

#### 3.1.8 GO and KEGG functional enrichment analysis results

**FIGURE 2:**
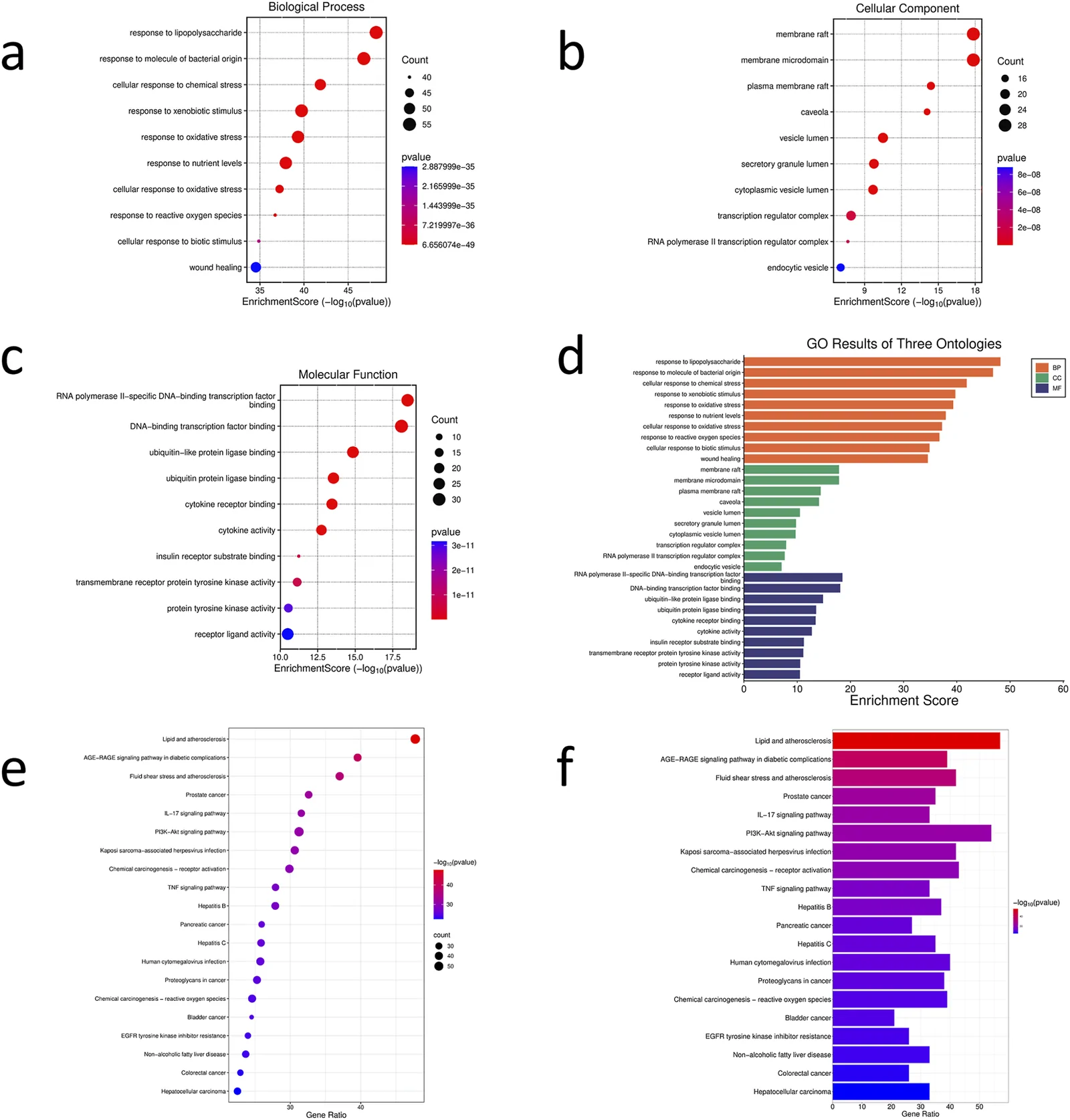
GO and KEGG functional enrichment analysis of the overlapping targets. **(a–c)** The most significantly enriched terms in the Gene Ontology (GO) categories: **(a)** biological process (BP), **(b)** cellular component (CC), and **(c)** molecular function (MF). **(d)** GO pathway enrichment analysis. **(e)** Bubble chart of significantly enriched KEGG pathways. **(f)** KEGG pathway enrichment analysis. Enriched terms were selected with *P*-value <0.05 and q-value <0.05. CON: control; MOD: model; QR: quercetin; SM: silymarin.

GO enrichment analysis revealed 3,312 significantly enriched terms across three ontological categories: Biological Process (BP, 2,943 terms), Cellular Component (CC, 115 terms), and Molecular Function (MF, 254 terms). Within the BP category, the five most significantly enriched terms were: response to lipopolysaccharide, response to molecule of bacterial origin, cellular response to chemical stress, response to xenobiotic stimulus, and response to oxidative stress (refer to Figure 2a). In the CC category, significant enrichment was detected for components including membrane raft, membrane microdomain, plasma membrane raft, caveola, and vesicle lumen (refer to Figure 2b). For MF, the most significantly enriched terms notably included DNA-binding transcription factor binding, along with ubiquitin-like protein ligase, ubiquitin protein ligase, and cytokine receptor-binding functions (Figure 2c). The chord plot in Figure 2d further illustrates the intricate relationships between these enriched GO terms and their associated genes, revealing the multi-level regulatory networks involved in quercetin’s anti-fibrotic mechanism (Figure 2d).

The KEGG pathway analysis identified 198 significantly enriched pathways, including Lipid and Atherosclerosis, AGE-RAGE Signaling Pathway in Diabetic Complications, Fluid Shear Stress and Atherosclerosis, Prostate Cancer, IL-17 Signaling Pathway, PI3K-Akt Signaling Pathway, Kaposi Sarcoma-Associated Herpesvirus Infection, Chemical Carcinogenesis-Receptor Activation, TNF Signaling Pathway, and Hepatitis B (Figures 2e,f) (Zhou et al., 2021; Xu et al., 2022). These pathways are intricately linked to processes such as inflammation, cancer, metabolism, and viral infection, suggesting that quercetin may exert its anti-fibrotic effects through modulation of these signaling cascades.

### 3.2 Effects of quercetin on the general status and body weight of liver fibrotic rats

Throughout the experimental period, rats in the CON group exhibited normal activity levels, smooth fur, stable food intake, and a consistent pattern of weight gain. Conversely, rats in the MOD group exhibited progressive lethargy, rough fur, and reduced food consumption following CCl_4_ injections, which was accompanied by a significant retardation in weight gain, with some individuals experiencing weight loss. In the quercetin intervention (QR) group, rats demonstrated an enhanced overall condition relative to the model (MOD) group, as evidenced by increased activity levels, improved fur condition, higher food intake, and a reduced rate of weight loss.

At baseline, no significant differences in body weight were observed among the three groups at the commencement of the experiment (*P* > 0.05). By the conclusion of the eighth week, the body weights were recorded as (285.67 ± 15.34) g for the control (CON) group (235.45 ± 20.12) g for the MOD group, and (250.34 ± 18.56) g for the QR group. Statistical analysis revealed that the CON group exhibited a significantly higher body weight than the MOD group (*P* < 0.01). While the QR group showed a trend toward increased body weight compared to the MOD group, the difference was not statistically significant (*P* > 0.05). These findings suggest that quercetin partially mitigated the CCl_4_-induced weight loss and deterioration in general health in rats with liver fibrosis.

### 3.3 Histopathological observations

**FIGURE 3:**
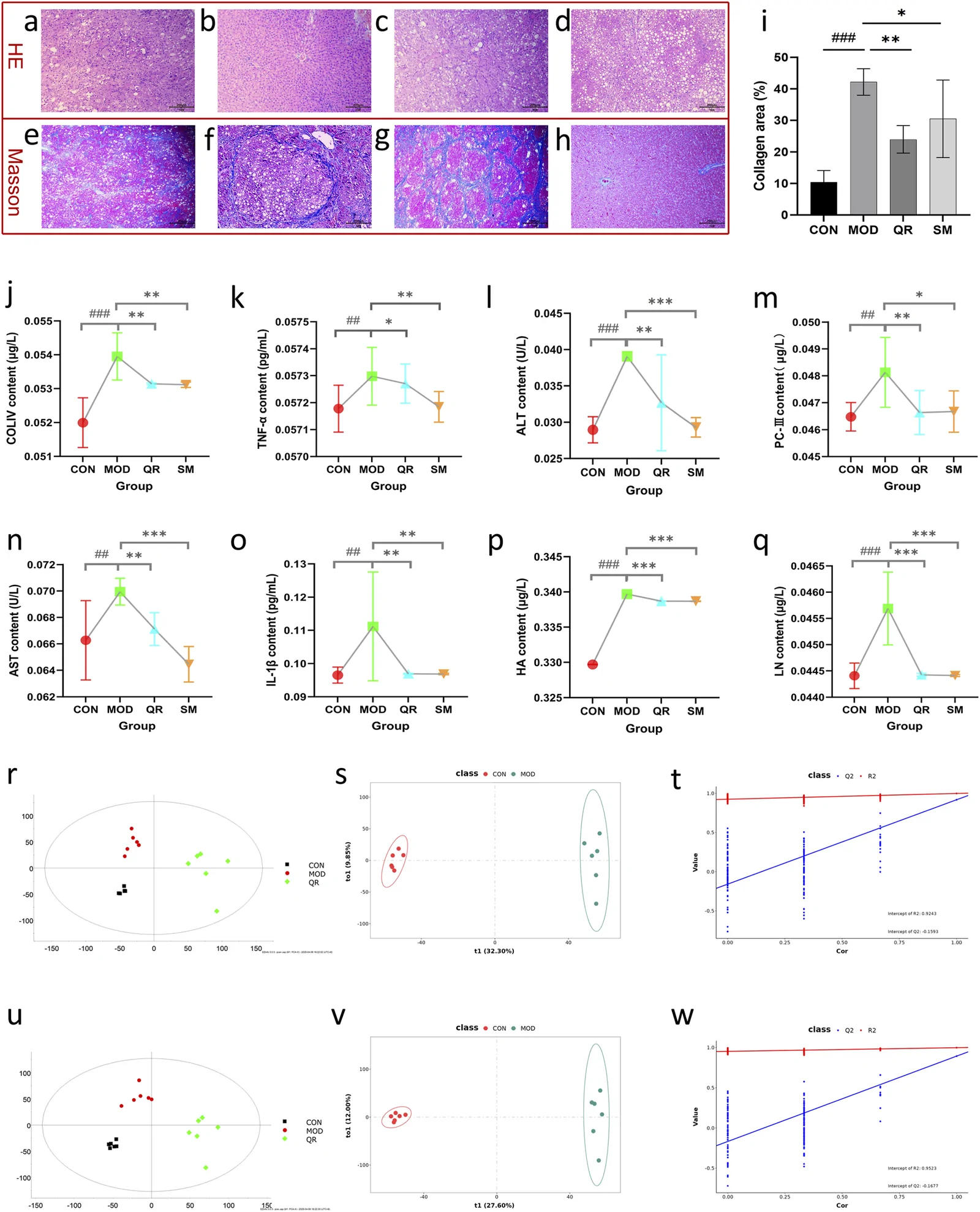
Quercetin attenuates CCl_4_-induced liver fibrosis and metabolic disorders in rats. **(a–h)** Histopathology of liver sections stained with H&E **(a–d)** and Masson’s trichrome **(e–h)** from CON **(a, e)**, MOD **(b,f)**, QR **(c,g)**, and SM **(d,h)** groups. Scale bar = 100 μm. **(i)** Quantitative analysis of collagen deposition area. Data are mean ± SD (n = 6). ###*P* < 0.001 vs. CON; **P < 0.01 vs. MOD; **P < 0.01 vs. CON; *P < 0.05 vs. CON (Tukey HSD). SM vs. MOD: *P* = 0.11 (not significant). **(j–q)** Effects of quercetin and silymarin on serum biochemical indicators, hepatic fibrosis markers, and inflammatory cytokines. Serum levels of COLIV **(j)**, TNF-α **(k)**, ALT **(l)**, PC-III **(m)**, AST **(n)**, IL-1β **(o)**, HA **(p)**, and LN **(q)**. Data are shown for CON, MOD, QR, and SM groups (*n* = 6 per group). #*P* < 0.05, ##*P* < 0.01, ###*P* < 0.001 vs. CON; *P < 0.05, **P < 0.01, ***P < 0.001 vs. MOD (one-way ANOVA with Tukey’s HSD *post hoc* test). CON: control group (corn oil); MOD: CCl_4_-induced model group; QR: quercetin 50 mg/kg/day; SM: silymarin 100 mg/kg/day. **(r–w)** Metabolomic profiles: PCA score plots in positive **(r)** and negative **(u)** modes; OPLS-DA score plots in positive **(s)** and negative **(v)** modes; permutation tests (200 iterations) for positive **(t)** and negative **(w)** modes. CON, control; MOD, model; QR, quercetin; SM, silymarin; PCA, principal component analysis; OPLS-DA, orthogonal projections to latent structures-discriminant analysis.

H&E staining demonstrated significant pathological differences in liver tissues across the various treatment groups. In the control (CON) group, the liver lobular architecture was well-preserved, with hepatocytes organized in orderly cords radiating from the central vein. The cell nuclei were uniform in both shape and staining, and there was no evident infiltration of inflammatory cells (Figure 3a). In contrast, the model (MOD) group demonstrated severely disrupted liver lobular architecture, characterized by marked hepatocyte swelling, degeneration, and vacuolization. The nuclei appeared irregular with condensed chromatin, and there was substantial infiltration of lymphocytes, monocytes, and neutrophils, both in the portal areas and within the liver lobules (Figure 3b).

The QR group exhibited significant amelioration of pathological damage relative to the MOD group. The liver lobular structure was partially restored, with reduced hepatocyte swelling and vacuolization, and a marked decrease in inflammatory cell infiltration, which was primarily confined to the portal areas (Figure 3c). The silymarin-treated (SM) group showed an intermediate level of pathological improvement between the QR and MOD groups, with partial restoration of liver lobular architecture and an approximately 35% reduction in inflammatory cell infiltration compared to the MOD group (Figure 3d). These findings suggest that quercetin intervention effectively attenuates the pathological progression of CCl_4_-induced liver fibrosis.

Masson’s trichrome staining revealed significant variations in collagen fiber distribution across the experimental groups. In the control (CON) group, collagen fibers were predominantly localized around the portal areas and central veins, appearing thin and delicate. There was minimal collagen deposition within the liver lobules, and the hepatic sinusoidal structures remained clear and intact (Figure 3e). In contrast, the model (MOD) group demonstrated pronounced collagen fiber proliferation in the liver tissue, characterized by a diffuse distribution forming thick fibrous bundles that partitioned the liver lobules, resulting in the formation of pseudolobules. Collagen around the portal areas and central veins was markedly thickened and increased, leading to compression, narrowing, or even occlusion of the hepatic sinusoids (Figure 3f).

The QR group exhibited a substantial reduction in collagen fiber deposition compared to the MOD group. The fibrous bundles exhibited a reduction in thickness, the formation of pseudolobules was diminished, and collagen proliferation surrounding the portal areas and central veins was mitigated, with partial restoration of the hepatic sinusoidal architecture (Figure 3g). In the SM group, the area of collagen deposition was reduced by 27.6% (*P* < 0.01) compared to the MOD group, although it remained higher than that observed in the QR group (Figure 3h).

Quantitative image analysis of Masson-stained sections confirmed these observations. The collagen-deposited area in the MOD group (42.18% ± 4.35%) was significantly greater than that in the CON group (10.40% ± 3.70%; *P* < 0.001). Quercetin treatment significantly reduced the collagen area to 23.99% ± 4.28% (*P* = 0.006 vs. MOD), representing a reduction of approximately 43.1%. Silymarin treatment reduced the collagen area to 30.52% ± 12.76%, representing a reduction of 27.6%, although the difference from the MOD group was not statistically significant (*P* = 0.11) (Figure 3i).

These findings indicate that quercetin effectively inhibited CCl_4_-induced hyperplasia and deposition of collagen fibers in hepatic tissue.

### 3.4 Biochemical indicator measurement results

The serum and liver tissue biochemical parameters are summarized in Figures 3j–q. Compared with the CON group, the MOD group showed significant elevations in all measured indicators (all *P* < 0.01). Specifically, serum ALT and AST levels in the MOD group were 0.03907 ± 0.00062 and 0.06968 ± 0.00071 (mean ± SD, *n* =6), respectively, versus 0.02895 ± 0.00180 and 0.06628 ± 0.00273 in the CON group.

Quercetin (QR) treatment significantly attenuated these CCl_4_-induced increases. In the QR group, ALT decreased to 0.03268 ± 0.00660 (*P* < 0.01 vs. MOD) and AST to 0.06661 ± 0.00247 (*P* < 0.05 vs. MOD). Similarly, QR significantly reduced the hepatic fibrosis markers COLIV, PC-III, HA, and LN, as well as the inflammatory cytokines TNF-α and IL-1β (all *P* < 0.05 or *P* < 0.01 vs. MOD).

The positive control silymarin (SM) group also effectively alleviated the CCl_4_-induced biochemical alterations. Compared with the MOD group, SM treatment significantly reduced serum ALT to 0.02930 ± 0.00134 (*P* < 0.01) and AST to 0.06445 ± 0.00134 (*P* < 0.05). The levels of COLIV, PC-III, HA, and LN in the SM group were also significantly lower than those in the MOD group (all *P* < 0.05). Moreover, SM markedly lowered hepatic TNF-α (0.05721 ± 0.00005 vs. 0.05730 ± 0.00009 in MOD, *P* < 0.05) and IL-1β (0.09686 ± 0.00021 vs. 0.11115 ± 0.01588 in MOD, *P* < 0.05). The detailed quantitative data for all four groups are presented in Figures 3j–q.

### 3.5 Metabolomics analysis results

Serum metabolites from the rats were systematically profiled by UPLC-QTOF-MS in both positive and negative ionization modes. As illustrated in Figures 3r,u, the unsupervised Principal Component Analysis (PCA) demonstrated clear segregation and distinct clustering among the CON, MOD, and QR groups in the score plots. Notably, the MOD group exhibited a significant deviation from the CON group, with a t (Roehlen et al., 2020) contribution exceeding 32%. In contrast, the metabolic profile of the QR group showed a tendency to revert towards the CON group profile. The tight clustering of sample points within each group (R^2^X > 0.85) suggested stable experimental procedures and high data quality, as indicated by a cluster radius of less than 15% in Figures 3r,u.

To further elucidate the regulatory effect of quercetin on the metabolic profile in liver fibrosis, a supervised OPLS-DA model was employed to enhance the identification of inter-group differences. In the positive ion mode, the CON and MOD groups were significantly separated along the t (Roehlen et al., 2020) axis, with an explained variance of 27.60% (*P* < 0.001), and the QR group was positioned between them (Figures 3s,t). A similar trend was observed in the negative ion mode, where the t (Roehlen et al., 2020) explained variance was 32.30%, with the metabolic profile of the QR group approaching that of the CON group (Figures 3v,w). The results of the permutation test corroborated the model’s substantial explanatory power (R^2^Y > 0.92) and statistical significance (*P* < 0.01), thereby affirming its reliability in classification.

In conclusion, both PCA and OPLS-DA analyses consistently demonstrated that liver fibrosis induced by carbon tetrachloride (CCl_4_) led to systemic metabolic disruptions (CON–MOD distance >40 units). Notably, intervention with quercetin significantly mitigated this effect, reducing the QR–CON distance by 60%, thus providing a foundation for further metabolic pathway analysis.

#### 3.5.1 Differential metabolite screening

Using criteria of VIP >1 and *P* < 0.05, we identified 108 differential metabolites between the CON and MOD groups. Of these, 54 were detected in positive ion mode (31 upregulated, 23 downregulated), while the remaining 54 were found in negative ion mode (42 upregulated, 12 downregulated). The differential metabolites were ranked in descending order based on their VIP values to highlight their significance in contribution (Table 2); a comprehensive list of metabolites is available in Appendix Table EV1. A total of 113 differential metabolites were identified between the MOD and QR groups using consistent screening criteria. In positive ion mode, 64 metabolites were altered (18 up, 46 down), while negative ion mode revealed 49 metabolites (24 up, 25 down). The ranked results are detailed in Table 3, while the comprehensive dataset is provided in Appendix Table EV2.

**TABLE 2:** Differential metabolites identified between the CON and MOD groups (Table content would follow).

No	Metabolites	Source	m/s	Retentiontimes [s]	MS1keggID	Ion mode	VIP	Regulated (M/C)	Regulated (Q/M)
1	S-(2-carboxypropyl)-cysteamine	Exogenous	198.04116	0.738,111,667	C06662	Esi-	1.72524	↑	↓
2	Indoleacetic acid	Exogenous	130.0662,595	3.567,216,667	C08313	Esi-	1.61922	↑	↓
3	Indoleacetic acid	Exogenous	174.0564,013	3.567,333,333	C00954	Esi-	1.58309	↑	↓
4	Androsterone	Exogenous	289.2,176,784	6.588,158,333	C05293	Esi-	1.55881	↓	​
5	Caffeoylferuloylspermidine	Exogenous	522.2,022,315	0.717,376,667	C01835	ESI+	1.51458	↑	​
6	Cortisol	Endogenous	363.216,225	3.961,766,667	C00735	ESI+	1.47876	↑	↓
7	7-Ketocholesterol	Endogenous	401.3,409,117	6.18735	C05455	ESI+	1.45291	↓	↑
8	Melibiose	Endogenous	360.1,492,495	0.6,980,975	C01083	ESI+	1.44914	↑	​
9	Linoleic acid	Endogenous	281.2,469,404	6.73615	C01595	ESI+	1.43826	↓	↓
10	Maltotriose	Endogenous	503.1,610,268	0.722,233,333	C01835	Esi-	1.43309	↑	​
11	Phosphatidylserine	Exogenous	401.1,299,896	0.70015	C01083	Esi-	1.42884	↑	​
12	Alpha-linolenic acid	Endogenous	279.2,316,138	4.909,283,333	C06426	ESI+	1.42447	↑	↓
13	Docosapentaenoic acid (22n-6)	Exogenous	329.2,492,544	7.190,066,667	C16513	Esi-	1.41397	↓	​
14	Cucurbic acid	Exogenous	211.1,343,533	3.738,616,667	C16309	Esi-	1.41296	↑	↓
15	Jasmonic acid	Exogenous	209.1,183,131	3.633,125	C08491	Esi-	1.40976	↑	​
16	Glucosamine	Exogenous	180.0874,318	1.289,394,167	C16675	ESI+	1.40955	↑	​
17	Ibervirin	Exogenous	165.0504,555	0.604,575	C02763	ESI+	1.40398	↑	​
18	11,12,15-Trihydroxyeicosatrienoic acid	Exogenous	355.2,453,414	3.890,066,667	C14811	ESI+	1.40025	↑	​
19	Norepinephrine	Endogenous	170.0807,032	1.629,873,333	C00314	ESI+	1.39925	↑	↓
20	Pregnenolone	Endogenous	315.2,336,982	6.7243	C04042	Esi-	1.39587	↑	​

**TABLE 3:** Differential metabolites identified between the MOD and QR groups (Table content would follow).

No	Metabolites	Source	m/s	Retentiontimes [s]	MS1keggID	Ion mode	VIP	Regulated (M/Q)
1	Histamine	Exogenous	112.0869,437	1.2546	C00388	ESI+	1.78051	↓
2	Cytosine	Endogenous	112.0504,326	1.119,461,667	C00380	ESI+	1.71795	↓
3	7-Ketocholesterol	Endogenous	401.3,409,169	6.587,725	C05455	ESI+	1.70105	↑
4	Pilocarpine	Exogenous	209.1,281,134	2.905,566,667	C07474	ESI+	1.68745	↑
5	Alpha-linolenic acid	Endogenous	279.2,317,359	5.684,625	C06426	ESI+	1.65011	↓
6	7-Ketocholesterol	Endogenous	401.3,409,117	6.18735	C05455	ESI+	1.64845	↑
7	Ergosterol	Endogenous	397.3,447,569	9.0278	C05441	ESI+	1.6484	↑
8	Norfuraneol	Exogenous	115.038979	3.279,033,333	C00429	ESI+	1.6405	↓
9	L-Proline	Endogenous	116.0704,866	2.993,191,667	C00148	ESI+	1.6403	↓
10	2-Phenylaminoadenosine	Exogenous	359.1,485,102	3.470,566,667	C10682	ESI+	1.63872	↓
11	3-Methylthiopropionic acid	Endogenous	119.0171,133	3.022,983,333	C08276	Esi-	1.63816	↑
12	N-Acetylhistamine	Endogenous	152.0827,405	1.23073	C05135	Esi-	1.63599	↓
13	p-Cresol	Endogenous	107.0501,713	3.150,541,667	C00556	Esi-	1.6329	↓
14	4-Pyridoxic acid	Exogenous	184.0599,777	2.699,233,333	C00847	ESI+	1.62062	↓
15	Adrenic acid	Endogenous	331.2,646,598	7.545,733,333	C16527	Esi-	1.61935	↑
16	3-Hydroxymonoethylglycinexylidide	Endogenous	223.1,438,929	2.916,691,667	C16572	ESI+	1.61586	↑
17	13 S-hydroxyoctadecadienoic acid	Endogenous	297.241,804	5.6376	C14825	ESI+	1.6116	↓
18	7-Ketocholesterol	Endogenous	401.3,410,085	8.250,475	C05455	ESI+	1.59388	↑
19	Indoleacetic acid	Exogenous	174.0564,013	3.567,333,333	C00954	Esi-	1.58679	↓
20	7-Azaindolizine	Exogenous	136.0867,262	1.214,295	C00147	ESI+	1.58567	↓

**FIGURE 4:**
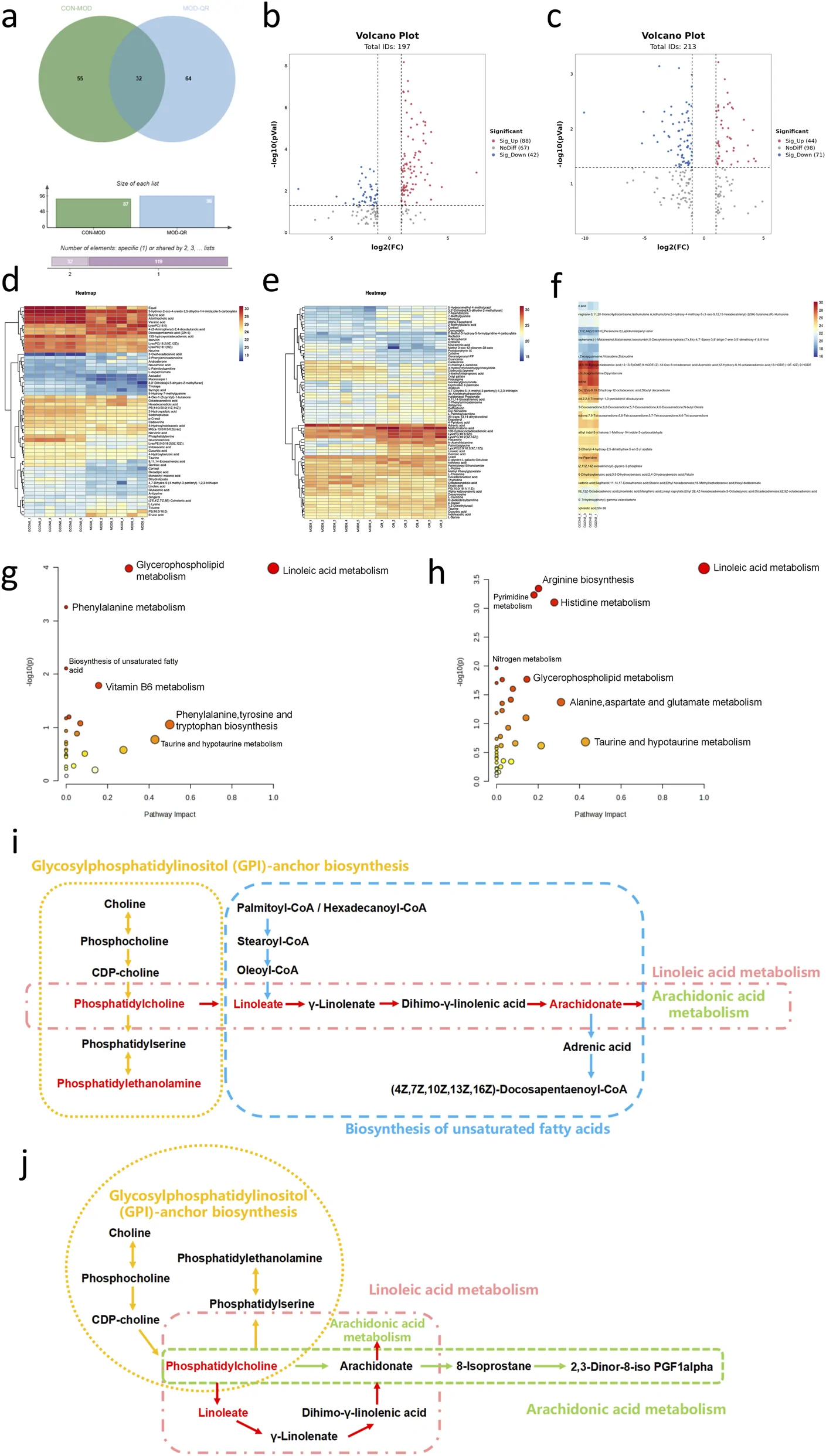
Quercetin reverses metabolic perturbations in CCl_4_-induced liver fibrosis. **(a)** Categorization of 32 potential biomarker metabolites reversed by quercetin. **(b,c)** Volcano plots of differential metabolites between CON and MOD groups in negative **(b)** and positive **(c)** modes (VIP >1.0, *P* < 0.05, FDR-adjusted q < 0.05). **(d)** Heatmap of differential metabolites between CON and MOD groups. **(e)** Heatmap of differential metabolites between MOD and QR groups. **(f)** Heatmap of the 32 overlapping metabolites across CON, MOD, and QR groups. **(g)** Altered metabolic pathways in MOD vs. CON identified by pathway analysis. **(h)** Altered metabolic pathways in MOD vs. QR. **(i)** Schematic of glycerophospholipid metabolism and GPI-anchor biosynthesis. **(j)** Schematic of linoleic acid and arachidonic acid metabolism. Metabolites were identified by UPLC-QTOF-MS (n = 6 per group). CON, control; MOD, CCl_4_-induced model; QR, quercetin 50 mg/kg/day; GPI, glycosylphosphatidylinositol.

Venn diagram analysis further compared the differential metabolite distributions between the CON–MOD and MOD–QR groups, revealing an overlap of 32 metabolites (Figure 4a). This finding suggests that quercetin intervention significantly reversed the aberrant expression of metabolites associated with liver fibrogenesis.

To visualize the differential metabolite abundance between the CON and MOD groups, volcano plot analysis was employed (Figures 4b,c). Applying a threshold of *P* < 0.05 and fold change (FC) > 1.5, we identified differential metabolites as follows. In the CON vs. MOD comparison, 73 metabolites were significantly upregulated and 35 downregulated (total 108). In the MOD vs. QR comparison, 42 metabolites were upregulated and 70 downregulated (total 112), while 190 metabolites showed no significant change. In the MOD group, saturated fatty acids like palmitic and stearic acids were notably increased (log_2_FC > 1), while TCA cycle intermediates such as succinic and malic acids were notably decreased (log_2_FC < −1). After QR intervention, volcano plots indicated that some metabolite levels shifted back towards those in the CON group.

Differential Metabolite Classification: The differential metabolites were mainly enriched in pathways associated with fatty acid, amino acid, and energy metabolism. Heatmap analysis revealed that 38 metabolites had significantly reversed expression levels in the QR group compared to the MOD group. Additionally, some metabolites that were significantly reduced between the CON and MOD groups showed a significant rise between the MOD and QR groups. Overall, quercetin intervention markedly reversed the metabolic disruptions in the liver fibrosis model (Figures 4d–f).

#### 3.5.2 Metabolic pathway enrichment analysis

Pearson correlation analysis and KEGG enrichment analysis were conducted on the 32 differential metabolites. The metabolites differing between the CON and MOD groups predominantly comprised phosphatidylcholines, phosphatidylethanolamines, linoleic acid, and arachidonic acid. These metabolites were significantly enriched in pathways intricately linked to the pathogenesis of liver fibrosis, including linoleic acid metabolism, glycerophospholipid metabolism, phenylalanine metabolism, vitamin B6 metabolism, unsaturated fatty acid biosynthesis, and glycosylphosphatidylinositol (GPI)-anchor biosynthesis (Figures 4g,i).

Differential metabolites between the MOD and QR groups were predominantly phosphatidylcholines and linolenates, among others. Post-quercetin intervention, significant alterations were observed in pathways such as linoleic acid metabolism, histidine metabolism, arachidonic acid metabolism, arginine biosynthesis, and GPI-anchor biosynthesis (Figures 4h,j). These pathways are intricately associated with the release of inflammatory cytokines, systemic antioxidant capacity, and overall metabolic regulation, indicating that quercetin may exert its anti-fibrotic effects through diverse metabolic mechanisms.

### 3.6 Molecular docking results

**FIGURE 5:**
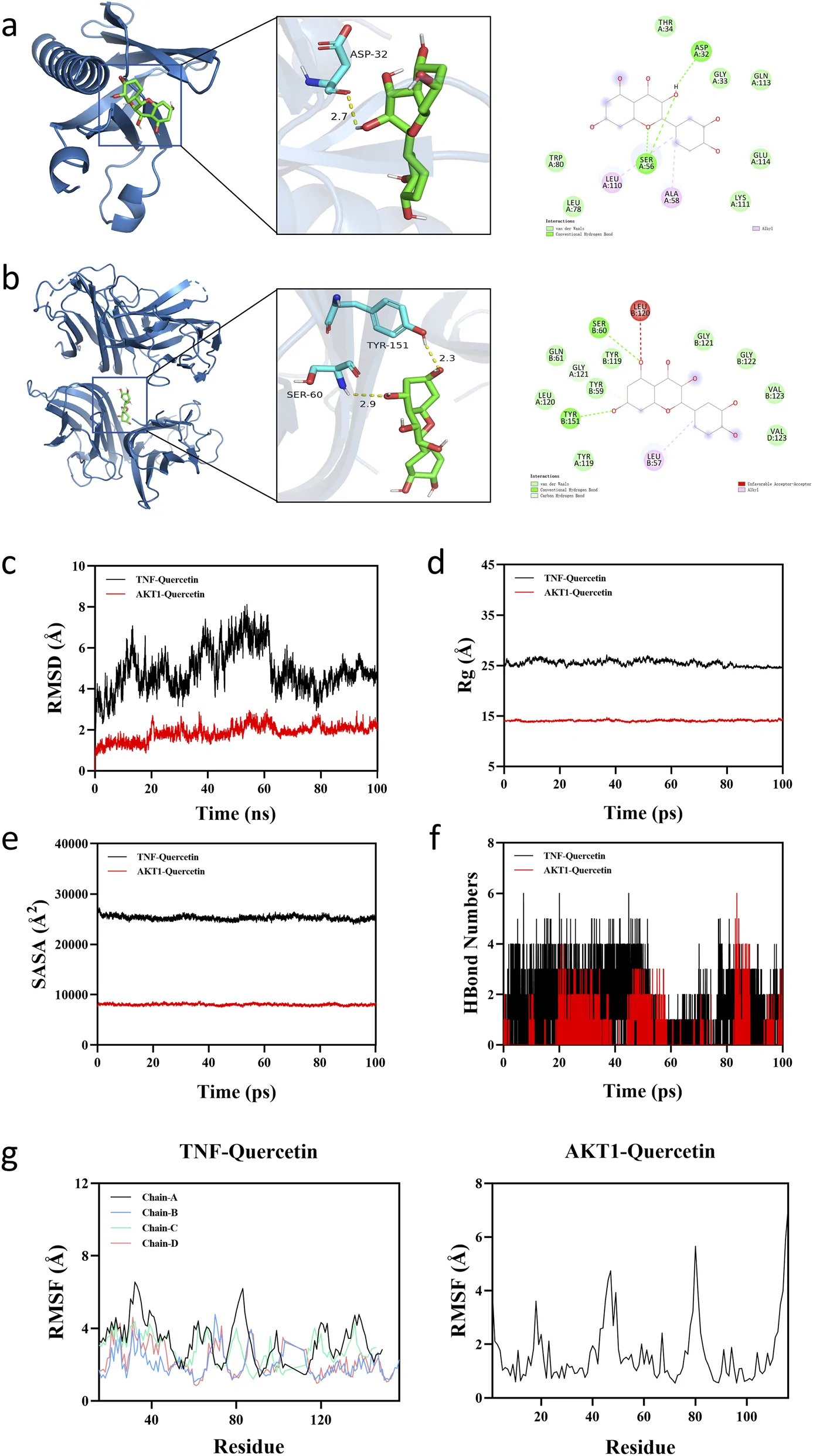
Molecular docking and dynamics simulations of quercetin with core targets. **(a, b)** Molecular docking of quercetin with AKT1 (PDB: 1H10) **(a)** and TNF (PDB: 2AZ5) **(b)** using AutoDock Vina. Left panels: 3D interaction diagrams; right panels: 2D interaction diagrams. Binding energies: −5.8 kcal/mol (AKT1) and −7.2 kcal/mol (TNF). Hydrogen bonds (green), hydrophobic interactions (pink), and van der Waals forces (light blue) are shown. **(c–g)** Molecular dynamics simulations over 100 ns using GROMACS 2022 (AMBER14SB force field) showing: **(c)** root-mean-square deviation (RMSD) of protein backbones; **(d)** radius of gyration (Rg); **(e)** solvent-accessible surface area (SASA); **(f)** number of hydrogen bonds between quercetin and the protein; **(g)** root-mean-square fluctuation (RMSF) per residue. All simulations were performed in triplicate with a 2 fs time step at 310 K and 1 bar.

Molecular docking analysis predicted that quercetin may bind with favorable affinity to the core targets AKT1 and TNF. The binding energy for the AKT1-quercetin complex was determined to be −5.8 kcal/mol. Within the active site of AKT1, the residues LEU110 and ALA58 were involved in hydrophobic interactions with quercetin, while ASP32 and SER56 participated in hydrogen bonding. Additionally, the residues TRP80, LEU78, LYS111, GLU114, GLY33, and GLN113 contributed van der Waals forces (Figure 5a). For the TNF-quercetin complex, the binding energy was calculated to be −7.2 kcal/mol. In the TNF binding site, the residue LEU57 engaged in hydrophobic interactions, whereas SER60 and TYR151 formed hydrogen bonds, and the residues LEU120, TYR119, VAL123, GLY122, and GLN61 provided van der Waals interactions (Figure 5b). These findings suggest that quercetin can stably bind within the active regions of both AKT1 and TNF, potentially modulating their biological functions.

### 3.7 Molecular dynamics simulation analysis

To assess the dynamic stability of quercetin-target protein complexes, molecular dynamics (MD) simulations were conducted over a duration of 100 nanoseconds. Analysis of the root mean square deviation (RMSD) demonstrated that the TNF-quercetin complex attained equilibrium at approximately 80 nanoseconds, exhibiting final fluctuations around 7.4 Å. In contrast, the AKT1-quercetin complex also reached equilibrium after 80 nanoseconds, with fluctuations around 2.2 Å (Figure 5c). The comparatively lower RMSD values observed for the AKT1 complex suggest enhanced conformational stability.

Further analysis of the radius of gyration (Rg) revealed minor fluctuations in the TNF-quercetin complex throughout the simulation period, whereas the AKT1-quercetin complex maintained relatively stable Rg values (Figure 5d). This stability implies superior structural integrity and the absence of significant conformational expansion or contraction in the AKT1 complex. Additionally, solvent accessible surface area (SASA) analysis indicated minor fluctuations in both complexes (Figure 5e), suggesting that quercetin binding modulates the microenvironments of the target proteins.

The analysis of hydrogen bonding revealed that the TNF-quercetin complex formed an average of approximately three hydrogen bonds, in contrast to the AKT1-quercetin complex, which formed about two (Figure 5f). This indicates stable hydrogen bond interactions between the ligand and the proteins. In the root mean square fluctuation (RMSF) analysis, most residues in both complexes exhibited RMSF values below 5 Å (Figure 5g), indicating low flexibility of the amino acid side chains and overall stability of the complex structures.

### 3.8 qPCR verification of key genes in the PI3K-Akt signaling pathway

**FIGURE 6:**
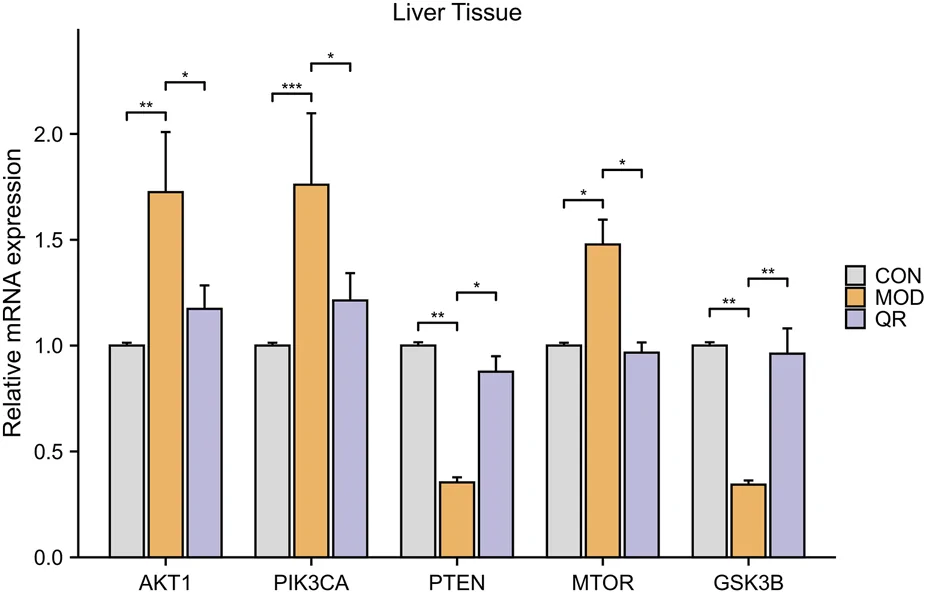
Quantitative real-time PCR analysis of PI3K-Akt signaling pathway-related genes (*AKT1*, *PIK3CA*, *PTEN*, *MTOR*, *GSK3B*) in the liver tissues of the CON, MOD, and QR groups. GAPDH was used as an endogenous control. Data are expressed as mean ± SD (n = 6 per group). Significance levels were set at *P < 0.05, **P < 0.01, and ***P < 0.001.

To experimentally substantiate the core targets predicted by network pharmacology, the mRNA expression levels of five pivotal genes within the PI3K-Akt signaling pathway—namely, *AKT1*, *PIK3CA*, *PTEN*, *MTOR*, and *GSK3B*—were quantified in liver tissues utilizing quantitative PCR (qPCR). The findings are depicted in Figure 6.

As illustrated in Figure 6, in comparison to the control (CON) group, the model (MOD) group demonstrated a significant upregulation in the mRNA expression of *AKT1*, *PIK3CA*, and *MTOR* (*P* < 0.01), whereas the expression levels of *PTEN* and *GSK3B* were markedly downregulated (*P* < 0.01). These alterations align with the anticipated activation of the pro-fibrotic PI3K-Akt signaling pathway in the CCl_4_-induced liver fibrosis model. Importantly, treatment with quercetin (QR group) significantly ameliorated these aberrant expression patterns. Relative to the MOD group, the QR group exhibited a pronounced reduction in the expression of *AKT1*, *PIK3CA* and *MTOR* (*P* < 0.05), alongside a significant increase in the expression of *PTEN* and *GSK3B* (*P* < 0.05). These qPCR results furnish direct experimental evidence that quercetin intervention effectively attenuates the hyperactivation of the PI3K-Akt signaling pathway at the mRNA level, thereby robustly corroborating the predictions derived from network pharmacology.

## 4 Discussion

In this study, we integrated network pharmacology, *in vivo* experimentation, metabolomics, and molecular dynamics simulations to systematically investigate the anti-fibrotic mechanism of quercetin. Our findings demonstrate that quercetin exerts multi-target therapeutic effects against CCl_4_-induced liver fibrosis through predicted interactions with AKT1 and TNF, suppression of the PI3K-Akt and TNF signaling pathways, and reversal of fibrosis-associated metabolic reprogramming.

A direct comparison with earlier reports further highlights the novelty of our work. Previous studies on quercetin in liver fibrosis primarily validated single targets (e.g., TGF-β1 or NF-κB) using conventional biochemical assays (Banerjee et al., 2025; Guo et al., 2022), while neglecting to explore the polypharmacological network or the associated metabolic rewiring. Our study offers three distinct advances: (a) systematic prediction and experimental validation of multi-target modulation (PI3K-Akt and TNF pathways via qPCR), (b) the first metabolomics evidence showing that quercetin reverses fibrosis-associated metabolic disturbances, including 32 differential metabolites enriched in linoleic acid and glycerophospholipid metabolism, and (c) 100-ns molecular dynamics simulations confirming stable binding of quercetin to AKT1 and TNF, thereby providing atomic-level support for direct target engagement.

Network pharmacology identified 203 overlapping targets between quercetin and liver fibrosis. Among these, *AKT1* (degree = 165), *IL6* (159), *TP53* (158), and *TNF* (157) emerged as hub proteins in the PPI network, indicating their central roles in quercetin’s intervention. The MCC algorithm further prioritized *NFKB1*, *STAT3*, *HIF1A*, and *BCL2* among the top-30 core targets. KEGG enrichment analysis revealed significant enrichment of the PI3K-Akt and TNF signaling pathways, both of which are well-established drivers of hepatic stellate cell (HSC) activation and extracellular matrix (ECM) deposition (Wang et al., 2026; Li L. et al., 2023). These predictions suggest that quercetin may simultaneously modulate inflammatory and fibrogenic cascades at the network level. This predicted modulation was directly validated by our qPCR analysis. Specifically, we observed that CCl_4_ induction significantly upregulated the mRNA expression of pro-fibrotic genes within the PI3K-Akt pathway, including *AKT1*, *PIK3CA*, and *MTOR*, while downregulating the pathway suppressors *PTEN* and *GSK3B*. Treatment with quercetin (50 mg/kg/day) significantly reversed these changes in mRNA expression, restoring the expression levels of all five genes towards those observed in the CON group (Figure 6). These results provide robust experimental confirmation that quercetin exerts its anti-fibrotic effects, at least in part, by inhibiting the overactivation of the PI3K-Akt signaling axis at the mRNA level.

*In vivo*, quercetin (50 mg/kg/day for 8 weeks) significantly attenuated CCl_4_-induced liver injury. Compared with the model group, quercetin reduced serum ALT by 16.4% (*P* < 0.01) and AST by 4.4% (*P* < 0.05), and it decreased collagen area by 43.1% (*P* = 0.006) as quantified by computer-assisted image analysis of Masson-stained sections. Histopathological examination (H&E) showed marked amelioration of hepatocyte swelling, vacuolization, and inflammatory infiltration. Moreover, quercetin significantly lowered the hepatic levels of fibrosis markers (COLIV, PC-III, HA, LN) and pro-inflammatory cytokines (TNF-α and IL-1β) (all *P* < 0.05). These results directly validate the network-predicted inhibition of the TNF signaling pathway and are consistent with previous reports that quercetin inhibits HSC activation and TGF-β1 signaling (Banerjee et al., 2025).

Metabolomics provided a novel metabolic dimension. A total of 32 differential metabolites were reversed by quercetin intervention, with significant enrichment in glycerophospholipid metabolism, linoleic acid metabolism, and glycosylphosphatidylinositol (GPI)-anchor biosynthesis. Specifically, in the model group, saturated fatty acids (e.g., palmitic acid, stearic acid) were markedly increased (log_2_FC > 1), whereas TCA cycle intermediates (e.g., succinic acid, malic acid) were decreased (log_2_FC < −1). Quercetin treatment partially normalized these changes, suggesting restoration of lipid and energy homeostasis. The regulation of the arachidonic acid and linoleic acid pathways may further contribute to reducing pro-fibrotic lipid mediators that fuel inflammation and HSC activation (Baek et al., 2024).

The integration of network pharmacology and metabolomics provides a multi-omics mechanistic insight. Our network analysis prioritized AKT1 and TNF as core hub proteins, and KEGG enrichment highlighted the PI3K-Akt and TNF signaling pathways. Meanwhile, metabolomics revealed significant alterations in linoleic acid metabolism, glycerophospholipid metabolism, and GPI-anchor biosynthesis. These two layers of data are not independent; rather, they are functionally linked. AKT1, a central node in the insulin/IGF-1 signaling cascade, directly regulates lipid metabolism through downstream effectors such as SREBP1c and PPARα. Activation of the PI3K-Akt axis promotes SREBP1c-mediated transcription of fatty acid synthase (FASN) and stearoyl-CoA desaturase 1 (SCD1), enzymes critical for linoleic acid and long-chain fatty acid metabolism (Röhrig and Schulze, 2016; Liu et al., 2023). Thus, quercetin-induced modulation of AKT1 could explain the reversed levels of linoleic acid and glycerophospholipids observed in the QR group. Furthermore, TNF-α signaling is intimately connected to sphingolipid metabolism. TNF-α stimulates sphingosine kinase 1 (SphK1) and neutral sphingomyelinase, leading to increased ceramide and sphingosine-1-phosphate (S1P) production. Our metabolomics data showed that 7-ketocholesterol and linoleic acid derivatives were altered, which are known to be downstream of TNF-α-induced oxidative stress. The correction of these metabolites by quercetin treatment is consistent with its suppression of TNF-α levels and the TNF signaling pathway. Together, these integrated findings suggest that quercetin attenuates liver fibrosis by simultaneously targeting PI3K-Akt/TNF signaling and the associated metabolic reprogramming of lipid and amino acid pathways.

Molecular docking revealed strong binding affinities of quercetin for AKT1 (−5.8 kcal/mol) and TNF (−7.2 kcal/mol). The docking poses showed that quercetin formed hydrogen bonds with ASP32 and SER56 in AKT1, and with SER60 and TYR151 in TNF. Molecular dynamics simulations over 100 ns confirmed the stability of both complexes: the AKT1-quercetin complex maintained a low RMSD (∼2.2 Å) and stable radius of gyration (Rg), while the TNF-quercetin complex exhibited slightly higher fluctuations (RMSD ∼7.4 Å) but remained stable after 80 ns? Both complexes retained an average of 2–3 hydrogen bonds throughout the simulation, and root-mean-square fluctuation (RMSF) analysis indicated low residue flexibility (<5 Å). These data suggest that quercetin may directly interact with AKT1 and TNF at the atomic level, but experimental validation is required to confirm actual binding. Molecular docking and MD simulations yield predictive insights that support but do not conclusively prove direct physical interactions. Experimental methods such as surface plasmon resonance (SPR) or isothermal titration calorimetry (ITC) would be required for confirmation.

We acknowledge that protein-level validation (e.g., Western blotting for AKT1 or other key targets) was not performed in this study, as the available liver tissue had been fully consumed by histological, biochemical, and qPCR analyses. This limits direct confirmation of whether the observed metabolic changes correspond to altered protein expression. Nevertheless, the convergence of network pharmacology predictions, qPCR results, and functional readouts (histology, serum biomarkers, metabolomics) provides converging evidence supporting the biological relevance of the identified PI3K-Akt and TNF pathway nodes. Future studies incorporating targeted protein expression analyses of these hub genes (e.g., AKT1, PIK3CA, PTEN, MTOR) would further substantiate the proposed mechanistic model.

Additionally, our metabolomics analysis was limited to serum samples; metabolomic profiling of liver tissue would yield complementary insights into intrahepatic metabolic alterations and merits investigation in future studies.

In summary, the convergence of evidence from network pharmacology, animal experiments, metabolomics, and molecular dynamics simulations strengthens the conclusion that quercetin acts through a synergistic, multi-target network against liver fibrosis. This integrative approach provides a robust model for elucidating the polypharmacology of natural products and offers a mechanistic foundation for considering quercetin as a promising lead compound for anti-fibrotic drug development. In direct comparison with the positive control silymarin, quercetin demonstrated comparable or slightly superior effects in reducing collagen deposition (43.1% vs. 27.6% for silymarin) and in lowering key inflammatory markers such as TNF-α and IL-1β. These findings suggest that quercetin not only matches the efficacy of a standard reference agent but may offer additional metabolic regulatory benefits through its modulation of lipid and amino acid pathways.

## 5 Conclusion

Quercetin exerts anti-fibrotic effects through a multi-target mechanism involving direct interaction with core targets like AKT1 and TNF, inhibition of key signaling pathways (PI3K-Akt, TNF), and reversal of fibrosis-associated metabolic disturbances. This study provides a comprehensive mechanistic foundation for considering quercetin as a potential therapeutic agent for liver fibrosis.

## Data Availability

The datasets generated and/or analyzed during the current study are publicly available. These data can be found here: https://www.ncbi.nlm.nih.gov/bioproject/PRJNA992181/.
