# Supplementary material for: Molecular and metabolic mechanisms of quercetin in liver fibrosis: an integrated network pharmacology, metabolomics, and molecular dynamics study
Source: Front Pharmacol. 2026 Aug 27;17:1838052. doi: 10.3389/fphar.2026.1838052 (PMC13563204; doi:10.3389/fphar.2026.1838052)
Supplement: Supplementary file 1 [file Supplementaryfile1.docx]

Supplementary material：

| No | Metabolites | Source | m/s | Retentiontimes  [s] | MS1keggID | Ionmode | VIP | Regulated (M/C) | Regulated (Q/M) |
| --- | --- | --- | --- | --- | --- | --- | --- | --- | --- |
| 1 | S-(2-carboxypropyl)-Cysteamine | Exogenous | 198.04116 | 0.738111667 | C06662 | ESI- | 1.72524 | ↑ | ↓ |
| 2 | Indoleacetic acid | Exogenous | 130.0662595 | 3.567216667 | C08313 | ESI- | 1.61922 | ↑ | ↓ |
| 3 | Indoleacetic acid | Exogenous | 174.0564013 | 3.567333333 | C00954 | ESI- | 1.58309 | ↑ | ↓ |
| 4 | Androsterone | Exogenous | 289.2176784 | 6.588158333 | C05293 | ESI- | 1.55881 | ↓ |  |
| 5 | Caffeoylferuloylspermidine | Exogenous | 522.2022315 | 0.717376667 | C01835 | ESI+ | 1.51458 | ↑ |  |
| 6 | Cortisol | Endogenous | 363.216225 | 3.961766667 | C00735 | ESI+ | 1.47876 | ↑ | ↓ |
| 7 | 7-Ketocholesterol | Endogenous | 401.3409117 | 6.18735 | C05455 | ESI+ | 1.45291 | ↓ | ↑ |
| 8 | Melibiose | Endogenous | 360.1492495 | 0.6980975 | C01083 | ESI+ | 1.44914 | ↑ |  |
| 9 | Linoleic acid | Endogenous | 281.2469404 | 6.73615 | C01595 | ESI+ | 1.43826 | ↓ | ↓ |
| 10 | Maltotriose | Endogenous | 503.1610268 | 0.722233333 | C01835 | ESI- | 1.43309 | ↑ |  |
| 11 | Phosphatidylserine | Exogenous | 401.1299896 | 0.70015 | C01083 | ESI- | 1.42884 | ↑ |  |
| 12 | Alpha-Linolenic acid | Endogenous | 279.2316138 | 4.909283333 | C06426 | ESI+ | 1.42447 | ↑ | ↓ |
| 13 | Docosapentaenoic acid (22n-6) | Exogenous | 329.2492544 | 7.190066667 | C16513 | ESI- | 1.41397 | ↓ |  |
| 14 | Cucurbic acid | Exogenous | 211.1343533 | 3.738616667 | C16309 | ESI- | 1.41296 | ↑ | ↓ |
| 15 | Jasmonic acid | Exogenous | 209.1183131 | 3.633125 | C08491 | ESI- | 1.40976 | ↑ |  |
| 16 | Glucosamine | Exogenous | 180.0874318 | 1.289394167 | C16675 | ESI+ | 1.40955 | ↑ |  |
| 17 | Ibervirin | Exogenous | 165.0504555 | 0.604575 | C02763 | ESI+ | 1.40398 | ↑ |  |
| 18 | 11,12,15-trihydroxyeicosatrienoic acid | Exogenous | 355.2453414 | 3.890066667 | C14811 | ESI+ | 1.40025 | ↑ |  |
| 19 | Norepinephrine | Endogenous | 170.0807032 | 1.629873333 | C00314 | ESI+ | 1.39925 | ↑ | ↓ |
| 20 | Pregnenolone | Endogenous | 315.2336982 | 6.7243 | C04042 | ESI- | 1.39587 | ↑ |  |
| 21 | Melibiose | Endogenous | 360.149284 | 1.124598333 | C01083 | ESI+ | 1.3905 | ↑ |  |
| 22 | Taurine | Endogenous | 126.021692 | 0.603380833 | C00245 | ESI+ | 1.38482 | ↑ | ↓ |
| 23 | 5-hydroxy-2-oxo-4-ureido-2,5-dihydro-1H-imidazole-5-carboxylate | Exogenous | 201.0253171 | 0.605400833 | C00841 | ESI- | 1.38193 | ↑ |  |
| 24 | Linoleic acid | Endogenous | 281.2468534 | 7.463966667 | C01595 | ESI+ | 1.37664 | ↓ | ↓ |
| 25 | Gentisic acid | Exogenous | 153.0191753 | 3.248108333 | C00230 | ESI- | 1.37393 | ↑ | ↓ |
| 26 | MG(a-13:0/0:0/0:0)[rac] | Endogenous | 289.2372847 | 3.88255 | C15492 | ESI+ | 1.36611 | ↓ |  |
| 27 | 7-Ketocholesterol | Endogenous | 401.3409138 | 9.04785 | C05455 | ESI+ | 1.36156 | ↓ | ↑ |
| 28 | L-Palmitoylcarnitine | Endogenous | 400.3414895 | 4.639466667 | C02301 | ESI+ | 1.36155 | ↓ | ↓ |
| 29 | 13S-hydroxyoctadecadienoic acid | Endogenous | 297.2417914 | 7.736066667 | C14767 | ESI+ | 1.3587 | ↓ | ↓ |
| 30 | Antipyrine | Exogenous | 223.0611554 | 3.363716667 | C00482 | ESI- | 1.35412 | ↑ | ↓ |
| 31 | PC(15:0/18:4(6Z,9Z,12Z,15Z)) | Exogenous | 738.5080416 | 6.79595 | C00350 | ESI- | 1.35384 | ↑ |  |
| 32 | Varanic acid | Endogenous | 435.3109684 | 5.4753 | C17658 | ESI- | 1.34943 | ↑ |  |
| 33 | MG(a-13:0/0:0/0:0)[rac] | Endogenous | 287.2232829 | 4.191425 | C08285 | ESI- | 1.34499 | ↓ |  |
| 34 | Gingerol | Exogenous | 295.1903402 | 4.144283333 | C10462 | ESI+ | 1.34282 | ↓ |  |
| 35 | 7-a,25-Dihydroxycholesterol | Endogenous | 417.3367331 | 7.376533333 | C15518 | ESI- | 1.34069 | ↓ | ↑ |
| 36 | L-Lysine | Exogenous | 169.0947313 | 0.688855 | C00534 | ESI+ | 1.33854 | ↓ | ↓ |
| 37 | 8-Hydroxy-7-methylguanine | Exogenous | 182.0658884 | 0.598661667 | C00082 | ESI+ | 1.33813 | ↑ |  |
| 38 | PC(15:0/18:3(6Z,9Z,12Z)) | Endogenous | 740.5239219 | 7.002791667 | C00350 | ESI- | 1.33705 | ↑ |  |
| 39 | 2-Hydroxyadipic acid | Endogenous | 161.0455899 | 0.695095833 | C02488 | ESI- | 1.33521 | ↑ |  |
| 40 | 2-Methyl-2-phenyl-undecane | Exogenous | 297.2417532 | 5.313633333 | C14767 | ESI+ | 1.33359 | ↓ |  |
| 41 | 7-Ketocholesterol | Endogenous | 401.3409146 | 8.7473 | C05455 | ESI+ | 1.32408 | ↓ | ↑ |
| 42 | Hexadecanedioic acid | Exogenous | 285.2077219 | 3.9815 | C19615 | ESI- | 1.3165 | ↓ | ↑ |
| 43 | Alpha-Linolenic acid | Endogenous | 279.2316427 | 7.949541667 | C06426 | ESI+ | 1.31255 | ↓ | ↓ |
| 44 | LysoPC(18:0) | Endogenous | 524.3689315 | 5.899958333 | C04230 | ESI+ | 1.31252 | ↓ |  |
| 45 | D-Glucose | Exogenous | 179.0564081 | 0.67333 | C01487 | ESI- | 1.31233 | ↑ |  |
| 46 | PE(14:0/22:5(4Z,7Z,10Z,13Z,16Z)) | Exogenous | 736.4918157 | 6.615525 | C00350 | ESI- | 1.30971 | ↑ |  |
| 47 | 8,11,14-Eicosatrienoic acid | Exogenous | 307.2628186 | 4.233058333 | C16522 | ESI+ | 1.30962 | ↑ | ↓ |
| 48 | Caffeoylferuloylspermidine | Exogenous | 522.2022558 | 1.183393333 | C01835 | ESI+ | 1.3091 | ↑ |  |
| 49 | 3-Methyluric acid | Exogenous | 181.0367467 | 2.659983333 | C16355 | ESI- | 1.29515 | ↓ |  |
| 50 | L-dopachromate |  | 208.0505597 | 3.0535 | C05596 | ESI- | 1.2915 | ↓ |  |
| 51 | Varanic acid | Endogenous | 435.3108844 | 5.838883333 | C17658 | ESI- | 1.2894 | ↑ |  |
| 52 | p-Cresol | Exogenous | 107.0501713 | 3.150541667 | C00556 | ESI- | 1.28882 | ↑ | ↓ |
| 53 | Butyric acid | Endogenous | 87.04493222 | 3.208966667 | C02632 | ESI- | 1.28844 | ↑ |  |
| 54 | 2-Phenylaminoadenosine | Exogenous | 359.1485102 | 3.470566667 | C10682 | ESI+ | 1.28722 | ↓ | ↓ |
| 55 | Linoleic acid | Endogenous | 281.2468051 | 4.183008333 | C01595 | ESI+ | 1.27937 | ↑ | ↓ |
| 56 | 13S-hydroxyoctadecadienoic acid | Endogenous | 297.2418135 | 4.3216 | C14767 | ESI+ | 1.27858 | ↓ | ↓ |
| 57 | Allolithocholic acid | Endogenous | 375.2894359 | 5.840433333 | C17658 | ESI- | 1.27803 | ↑ |  |
| 58 | Equol | Exogenous | 241.087237 | 3.897833333 | C14131 | ESI- | 1.27433 | ↑ |  |
| 59 | (2'E,4'Z,7'Z,8E)-Colnelenic acid | Endogenous | 293.2101636 | 5.020266667 | C16320 | ESI+ | 1.26291 | ↑ |  |
| 60 | Octadecanedioic acid | Endogenous | 315.2523028 | 4.317158333 | C14828 | ESI+ | 1.25588 | ↓ | ↓ |
| 61 | L-Palmitoylcarnitine | Exogenous | 400.3416411 | 5.71975 | C02990 | ESI+ | 1.25574 | ↓ | ↓ |
| 62 | Arachidonic acid | Endogenous | 305.2467002 | 4.50545 | C00219 | ESI+ | 1.25515 | ↑ |  |
| 63 | Eicosapentaenoic acid | Exogenous | 301.2171482 | 6.414366667 | C06428 | ESI- | 1.24942 | ↑ |  |
| 64 | PE(14:0/20:3(5Z,8Z,11Z)) | Exogenous | 712.4931157 | 6.776266667 | C00350 | ESI- | 1.24846 | ↑ |  |
| 65 | Oxoadipic acid | Exogenous | 159.0301766 | 0.729711667 | C00846 | ESI- | 1.24552 | ↑ |  |
| 66 | Thiotepa | Exogenous | 190.0558909 | 0.654015833 | C00624 | ESI+ | 1.23972 | ↑ | ↓ |
| 67 | LysoPC(18:2(9Z,12Z)) | Exogenous | 520.3383182 | 4.87845 | C04230 | ESI+ | 1.23486 | ↑ | ↓ |
| 68 | Traumatic acid | Endogenous | 227.1290908 | 3.76495 | C16308 | ESI- | 1.23363 | ↑ |  |
| 69 | Syringic acid | Exogenous | 197.0455365 | 3.161533333 | C10833 | ESI- | 1.23126 | ↑ |  |
| 70 | PS(16:0/16:0) | Endogenous | 734.5022577 | 6.678066667 | C02737 | ESI- | 1.2268 | ↓ |  |
| 71 | 4-(2-Aminophenyl)-2,4-dioxobutanoic acid | Exogenous | 208.0597245 | 3.051525 | C01252 | ESI+ | 1.22037 | ↓ |  |
| 72 | LysoPE(0:0/18:2(9Z,12Z)) | Exogenous | 478.2926246 | 4.830325 | C04438 | ESI+ | 1.2188 | ↑ | ↓ |
| 73 | 4-Hydroxybenzoic acid | Exogenous | 137.0244402 | 3.098491667 | C00156 | ESI- | 1.21666 | ↑ |  |
| 74 | 8,11,14-Eicosatrienoic acid | Exogenous | 307.2625408 | 4.457233333 | C16522 | ESI+ | 1.21361 | ↑ | ↓ |
| 75 | Monoethyl malonic acid | Exogenous | 155.0313544 | 1.028124167 | C00230 | ESI+ | 1.1906 | ↑ | ↓ |
| 76 | 4-Oxo-1-(3-pyridyl)-1-butanone | Exogenous | 162.0561755 | 2.950925 | C19567 | ESI- | 1.18861 | ↓ |  |
| 77 | Alpha-Linolenic acid | Endogenous | 279.2317359 | 5.684625 | C06426 | ESI+ | 1.18327 | ↑ | ↓ |
| 78 | PE(14:0/20:2(11Z,14Z)) | Exogenous | 714.5065043 | 6.975641667 | C00350 | ESI- | 1.18244 | ↑ |  |
| 79 | Gluconolactone | Endogenous | 177.0403901 | 0.691543333 | C01040 | ESI- | 1.17739 | ↑ |  |
| 80 | 5-Hydroxyindoleacetic acid | Endogenous | 192.0654181 | 5.54125 | C05635 | ESI+ | 1.17468 | ↑ |  |
| 81 | Ascladiol | Exogenous | 155.0350276 | 1.243285 | C03680 | ESI- | 1.16704 | ↑ | ↓ |
| 82 | Neurine | Endogenous | 104.1069245 | 0.696449167 | C00114 | ESI+ | 1.16175 | ↑ |  |
| 83 | Monoethyl malonic acid | Exogenous | 131.0350512 | 1.411325 | C00489 | ESI- | 1.15476 | ↑ | ↓ |
| 84 | Toluene | Exogenous | 91.05517775 | 3.489616667 | C01455 | ESI- | 1.15032 | ↓ |  |
| 85 | LysoPC(18:1(9Z)) | Exogenous | 522.3529865 | 5.372591667 | C04230 | ESI+ | 1.14873 | ↑ | ↓ |
| 86 | Jasmonic acid | Exogenous | 211.1325536 | 3.093083333 | C08491 | ESI+ | 1.12703 | ↓ |  |
| 87 | Macrocarpal I | Exogenous | 506.3116266 | 6.056333333 | C04438 | ESI- | 1.11623 | ↑ |  |
| 88 | Glutaconic acid | Endogenous | 129.0192741 | 0.689524167 | C02800 | ESI- | 1.11413 | ↑ |  |
| 89 | PC(14:0/20:2(11Z,14Z)) | Exogenous | 758.5684656 | 6.437116667 | C00157 | ESI+ | 1.11311 | ↑ |  |
| 90 | 4,7-Dihydro-5-(4-methyl-3-pentenyl)-1,2,3-trithiepin | Exogenous | 231.0361043 | 0.696449167 | C06677 | ESI- | 1.10941 | ↑ | ↓ |
| 91 | LysoPE(0:0/18:2(9Z,12Z)) | Exogenous | 476.277891 | 4.832983333 | C04438 | ESI- | 1.10778 | ↑ | ↓ |
| 92 | Neuraminic acid | Endogenous | 266.0892793 | 1.452508333 | C00212 | ESI- | 1.10263 | ↓ | ↑ |
| 93 | M-toluic Acid | Exogenous | 135.0451071 | 3.490025 | C07086 | ESI- | 1.09741 | ↓ |  |
| 94 | Monoethyl malonic acid | Exogenous | 131.0349975 | 1.038859167 | C00489 | ESI- | 1.09463 | ↑ | ↓ |
| 95 | 3-Oxohexadecanoic acid | Endogenous | 293.2085454 | 5.2674 | C16320 | ESI+ | 1.08947 | ↓ |  |
| 96 | Nervonic acid | Exogenous | 367.3555227 | 8.960858333 | C08323 | ESI+ | 1.08641 | ↓ | ↑ |
| 97 | 13S-hydroxyoctadecadienoic acid | Exogenous | 297.241804 | 5.6376 | C14825 | ESI+ | 1.08469 | ↑ | ↓ |
| 98 | Erucic acid | Exogenous | 339.3247446 | 7.875483333 | C08316 | ESI+ | 1.07155 | ↓ | ↑ |
| 99 | 7-Ketocholesterol | Endogenous | 401.3410085 | 8.250475 | C05455 | ESI+ | 1.0667 | ↓ | ↑ |
| 100 | LysoPE(0:0/20:4(5Z,8Z,11Z,14Z)) | Exogenous | 502.2906135 | 3.325933333 | C06999 | ESI+ | 1.06535 | ↑ |  |
| 101 | 3,3'-Dithiobis[4,5-dihydro-2-methylfuran] | Exogenous | 229.0377351 | 0.612728333 | C01424 | ESI- | 1.06434 | ↑ | ↓ |
| 102 | Sedoheptulose | Exogenous | 209.0665986 | 0.696736667 | C00309 | ESI- | 1.06305 | ↑ |  |
| 103 | Dihydrolipoate | Exogenous | 207.0508647 | 0.694951667 | C01087 | ESI- | 1.05336 | ↑ |  |
| 104 | Cadaverine | Endogenous | 103.1230283 | 0.80849 | C01746 | ESI+ | 1.04517 | ↑ |  |
| 105 | Otobanone | Exogenous | 339.1222084 | 4.397316667 | C01701 | ESI+ | 1.04 | ↑ |  |
| 106 | 3'-carboxy-alpha-chromanol | Exogenous | 105.0707647 | 3.754541667 | C06756 | ESI- | 1.0346 | ↑ |  |
| 107 | 3'-carboxy-alpha-chromanol | Exogenous | 149.0607007 | 3.754325 | C05629 | ESI- | 1.0231 | ↑ |  |
| 108 | Cortexolone | Exogenous | 347.2209028 | 4.512166667 | C02140 | ESI+ | 1.0131 | ↑ |  |

Table 1. Differential metabolites identified between the CON and MOD groups.

(Table content would follow)

| No | Metabolites | Source | m/s | Retentiontimes[s] | MS1keggID | lonmode | VIP | Regulated (M/Q) |
| --- | --- | --- | --- | --- | --- | --- | --- | --- |
| 1 | Histamine | Exogenous | 112.0869437 | 1.2546 | C00388 | ESI+ | 1.78051 | ↓ |
| 2 | Cytosine | Endogenous | 112.0504326 | 1.119461667 | C00380 | ESI+ | 1.71795 | ↓ |
| 3 | 7-Ketocholesterol | Endogenous | 401.3409169 | 6.587725 | C05455 | ESI+ | 1.70105 | ↑ |
| 4 | Pilocarpine | Exogenous | 209.1281134 | 2.905566667 | C07474 | ESI+ | 1.68745 | ↑ |
| 5 | Alpha-Linolenic acid | Endogenous | 279.2317359 | 5.684625 | C06426 | ESI+ | 1.65011 | ↓ |
| 6 | 7-Ketocholesterol | Endogenous | 401.3409117 | 6.18735 | C05455 | ESI+ | 1.64845 | ↑ |
| 7 | Ergosterol | Endogenous | 397.3447569 | 9.0278 | C05441 | ESI+ | 1.6484 | ↑ |
| 8 | Norfuraneol | Exogenous | 115.038979 | 3.279033333 | C00429 | ESI+ | 1.6405 | ↓ |
| 9 | L-Proline | Endogenous | 116.0704866 | 2.993191667 | C00148 | ESI+ | 1.6403 | ↓ |
| 10 | 2-Phenylaminoadenosine | Exogenous | 359.1485102 | 3.470566667 | C10682 | ESI+ | 1.63872 | ↓ |
| 11 | 3-Methylthiopropionic acid | Endogenous | 119.0171133 | 3.022983333 | C08276 | ESI- | 1.63816 | ↑ |
| 12 | N-Acetylhistamine | Endogenous | 152.0827405 | 1.23073 | C05135 | ESI- | 1.63599 | ↓ |
| 13 | p-Cresol | Endogenous | 107.0501713 | 3.150541667 | C00556 | ESI- | 1.6329 | ↓ |
| 14 | 4-Pyridoxic acid | Exogenous | 184.0599777 | 2.699233333 | C00847 | ESI+ | 1.62062 | ↓ |
| 15 | Adrenic acid | Endogenous | 331.2646598 | 7.545733333 | C16527 | ESI- | 1.61935 | ↑ |
| 16 | 3-Hydroxymonoethylglycinexylidide | Endogenous | 223.1438929 | 2.916691667 | C16572 | ESI+ | 1.61586 | ↑ |
| 17 | 13S-hydroxyoctadecadienoic acid | Endogenous | 297.241804 | 5.6376 | C14825 | ESI+ | 1.6116 | ↓ |
| 18 | 7-Ketocholesterol | Endogenous | 401.3410085 | 8.250475 | C05455 | ESI+ | 1.59388 | ↑ |
| 19 | Indoleacetic acid | Exogenous | 174.0564013 | 3.567333333 | C00954 | ESI- | 1.58679 | ↓ |
| 20 | 7-Azaindolizine | Exogenous | 136.0867262 | 1.214295 | C00147 | ESI+ | 1.58567 | ↓ |
| 21 | N-lactoyl-Glycine | Exogenous | 146.0457828 | 0.630328333 | C00025 | ESI- | 1.5767 | ↓ |
| 22 | L-Serine | Endogenous | 106.0498971 | 0.670063333 | C00065 | ESI+ | 1.56842 | ↓ |
| 23 | L-Lysine | Endogenous | 147.1127572 | 0.632365833 | C00047 | ESI+ | 1.56831 | ↓ |
| 24 | Linoleic acid | Endogenous | 281.2467851 | 5.416858333 | C01595 | ESI+ | 1.56273 | ↓ |
| 25 | Cytidine | Endogenous | 242.0785957 | 1.235158333 | C00475 | ESI- | 1.55938 | ↑ |
| 26 | Guanosine | Endogenous | 284.098225 | 2.403166667 | C00387 | ESI+ | 1.55341 | ↑ |
| 27 | Indoleacetic acid | Exogenous | 130.0662595 | 3.567216667 | C08313 | ESI- | 1.54331 | ↓ |
| 28 | Octadecanedioic acid | Endogenous | 315.2523041 | 4.505433333 | C14829 | ESI+ | 1.53308 | ↓ |
| 29 | 3,3'-Dithiobis[4,5-dihydro-2-methylfuran] | Exogenous | 229.0377351 | 0.612728333 | C01424 | ESI- | 1.53208 | ↓ |
| 30 | 3-Methyl-9H-carbazole-9-carboxaldehyde | Exogenous | 208.0774232 | 3.565683333 | C16604 | ESI- | 1.53181 | ↑ |
| 31 | 5-Hydroxymethyl-4-methyluracil | Exogenous | 155.0460083 | 1.339830833 | C03680 | ESI- | 1.52542 | ↓ |
| 32 | Norepinephrine | Endogenous | 170.0807032 | 1.629873333 | C00314 | ESI+ | 1.52395 | ↓ |
| 33 | Pilocarpine | Exogenous | 209.1281535 | 1.521085833 | C07474 | ESI+ | 1.51556 | ↑ |
| 34 | Neuraminic acid | Endogenous | 266.0892793 | 1.452508333 | C00212 | ESI- | 1.50646 | ↑ |
| 35 | Vanillic acid | Endogenous | 167.0352212 | 3.162733333 | C06672 | ESI- | 1.50294 | ↓ |
| 36 | 3b,15b,17a-Trihydroxy-pregnenone | Endogenous | 349.2348893 | 5.257866667 | C05475 | ESI+ | 1.49545 | ↓ |
| 37 | L-Threonine | Endogenous | 120.0655308 | 0.676918333 | C00188 | ESI+ | 1.4953 | ↓ |
| 38 | Cytidine | Endogenous | 244.0918559 | 1.229761667 | C00475 | ESI+ | 1.49497 | ↑ |
| 39 | 4,7-Dihydro-5-(4-methyl-3-pentenyl)-1,2,3-trithiepin | Exogenous | 231.0361043 | 0.696449167 | C06677 | ESI- | 1.49489 | ↓ |
| 40 | 7-Methylguanine | Exogenous | 188.0537441 | 2.363691667 | C06559 | ESI+ | 1.49032 | ↓ |
| 41 | Alpha-Tocopherol | Endogenous | 429.3732716 | 7.960775 | C02477 | ESI- | 1.48811 | ↓ |
| 42 | Isovalerylglucuronide | Exogenous | 265.1112904 | 0.766225833 | C00378 | ESI+ | 1.48485 | ↓ |
| 43 | 7-Ketocholesterol | Endogenous | 401.3409138 | 9.04785 | C05455 | ESI+ | 1.47415 | ↑ |
| 44 | Antipyrine | Exogenous | 223.0611554 | 3.363716667 | C00482 | ESI- | 1.47132 | ↓ |
| 45 | 8,11,14-Eicosatrienoic acid | Endogenous | 307.2628186 | 4.233058333 | C16522 | ESI+ | 1.46978 | ↓ |
| 46 | Hulupinic acid | Exogenous | 263.1289201 | 3.339908333 | C06082 | ESI- | 1.46746 | ↑ |
| 47 | All-trans-13,14-dihydroretinol | Endogenous | 289.2521834 | 3.962266667 | C15492 | ESI+ | 1.46438 | ↓ |
| 48 | Geranylgeranyl-PP | Endogenous | 449.1856798 | 3.683883333 | C00353 | ESI- | 1.46013 | ↑ |
| 49 | Methylmalonic acid | Endogenous | 117.0191412 | 0.7851325 | C00042 | ESI- | 1.45554 | ↓ |
| 50 | PC(14:1(9Z)/22:2(13Z,16Z)) | Exogenous | 784.5831306 | 6.437108333 | C00157 | ESI+ | 1.45513 | ↓ |
| 51 | 3,3',7-Trihydroxy-4'-methoxyflavone | Exogenous | 301.0702923 | 3.5599 | C10098 | ESI+ | 1.44633 | ↓ |
| 52 | 7-a,25-Dihydroxycholesterol | Endogenous | 417.3367331 | 7.376533333 | C15518 | ESI- | 1.44559 | ↑ |
| 53 | S-(2-carboxypropyl)-Cysteamine | Exogenous | 198.04116 | 0.738111667 | C06662 | ESI- | 1.44483 | ↓ |
| 54 | L-Palmitoylcarnitine | Endogenous | 400.3414895 | 4.639466667 | C02301 | ESI+ | 1.43814 | ↓ |
| 55 | Dethiobiotin | Exogenous | 127.0500944 | 2.892191667 | C00178 | ESI+ | 1.4332 | ↑ |
| 56 | 19-Norandrosterone | Endogenous | 277.215569 | 6.754016667 | C08362 | ESI+ | 1.43113 | ↓ |
| 57 | Methyl Phenylglyoxalate | Exogenous | 163.040065 | 3.341741667 | C12621 | ESI- | 1.41994 | ↓ |
| 58 | Uracil | Endogenous | 113.0344133 | 1.190886667 | C00106 | ESI+ | 1.41301 | ↑ |
| 59 | Aliskiren | Exogenous | 552.4018201 | 5.151316667 | C04230 | ESI+ | 1.40532 | ↓ |
| 60 | Cortisol | Endogenous | 363.2162716 | 4.398783333 | C00735 | ESI+ | 1.40025 | ↓ |
| 61 | Ascladiol | Exogenous | 155.0350276 | 1.243285 | C03680 | ESI- | 1.39805 | ↓ |
| 62 | Uracil | Endogenous | 113.034471 | 1.837675 | C00106 | ESI+ | 1.39636 | ↑ |
| 63 | Alpha-Linolenic acid | Endogenous | 279.2317161 | 7.07265 | C06426 | ESI+ | 1.39596 | ↓ |
| 64 | Methyl 3-oxo-12-oleanen-28-oate | Endogenous | 269.150623 | 2.977941667 | C00468 | ESI- | 1.39186 | ↑ |
| 65 | Gentisic acid | Exogenous | 153.0191753 | 3.248108333 | C00230 | ESI- | 1.39098 | ↓ |
| 66 | Genistein | Endogenous | 271.059905 | 3.871941667 | C06563 | ESI+ | 1.38964 | ↓ |
| 67 | 3b-Allotetrahydrocortisol | Endogenous | 365.2329138 | 3.720933333 | C05481 | ESI- | 1.38788 | ↑ |
| 68 | Histamine | Exogenous | 112.0869585 | 0.914848333 | C00388 | ESI+ | 1.3799 | ↓ |
| 69 | PG(16:0/18:1(11Z)) | Endogenous | 747.5183071 | 7.56645 | C00344 | ESI- | 1.37762 | ↑ |
| 70 | Alpha-Linolenic acid | Endogenous | 279.2317181 | 6.650808333 | C06426 | ESI+ | 1.3681 | ↓ |
| 71 | Osmundalin | Exogenous | 308.131427 | 3.24135 | C10738 | ESI+ | 1.36287 | ↓ |
| 72 | 7-Ketocholesterol | Endogenous | 401.3409146 | 8.7473 | C05455 | ESI+ | 1.36009 | ↑ |
| 73 | Gly-Norvaline | Exogenous | 175.1071811 | 0.73159 | C00062 | ESI+ | 1.34817 | ↓ |
| 74 | Thiotepa | Exogenous | 190.0558909 | 0.654015833 | C00624 | ESI+ | 1.34584 | ↓ |
| 75 | 2-Methylglutaric acid | Endogenous | 145.0505694 | 0.748506667 | C00064 | ESI- | 1.33937 | ↑ |
| 76 | 1,3-Dimethyluracil | Exogenous | 141.0656935 | 0.717414167 | C05828 | ESI+ | 1.33626 | ↓ |
| 77 | Adrenic acid | Endogenous | 331.26468 | 7.810866667 | C16527 | ESI- | 1.33555 | ↑ |
| 78 | trans-Ferulic acid | Exogenous | 193.0507442 | 3.384508333 | C01494 | ESI- | 1.33099 | ↓ |
| 79 | LysoPE(0:0/18:2(9Z,12Z)) | Exogenous | 478.2926246 | 4.830325 | C04438 | ESI+ | 1.32802 | ↓ |
| 80 | Nervonic acid | Exogenous | 367.3555227 | 8.960858333 | C08323 | ESI+ | 1.32089 | ↑ |
| 81 | Halobetasol Propionate | Exogenous | 243.0970795 | 2.900883333 | C00214 | ESI+ | 1.31969 | ↑ |
| 82 | LysoPC(18:1(9Z)) | Endogenous | 522.3529865 | 5.372591667 | C04230 | ESI+ | 1.31855 | ↓ |
| 83 | 24,25-Dihydroxyvitamin D | Exogenous | 415.3224189 | 6.856566667 | C17333 | ESI- | 1.30759 | ↑ |
| 84 | Taurine | Endogenous | 126.021692 | 0.603380833 | C00245 | ESI+ | 1.3053 | ↓ |
| 85 | Thymidine | Endogenous | 241.082781 | 2.877425 | C14131 | ESI- | 1.30117 | ↑ |
| 86 | Protoporphyrin IX | Endogenous | 563.2643749 | 6.0257 | C02191 | ESI+ | 1.29788 | ↑ |
| 87 | D-glycero-L-galacto-Octulose | Exogenous | 239.0776095 | 0.697709167 | C01487 | ESI- | 1.29745 | ↓ |
| 88 | Thyrotropin releasing hormone | Exogenous | 361.1656963 | 3.642216667 | C18167 | ESI- | 1.2952 | ↓ |
| 89 | Erucic acid | Exogenous | 339.3247446 | 7.875483333 | C08316 | ESI+ | 1.29511 | ↑ |
| 90 | Dibenzo[a,l]pyrene | Exogenous | 301.1042317 | 2.878666667 | C14131 | ESI- | 1.29235 | ↑ |
| 91 | Hexadecanedioic acid | Endogenous | 285.2076349 | 4.629166667 | C19615 | ESI- | 1.2905 | ↑ |
| 92 | Methyl levulinate | Exogenous | 129.055518 | 2.896833333 | C06102 | ESI- | 1.28795 | ↑ |
| 93 | L-Carnitine | Endogenous | 162.1120026 | 0.700583333 | C00318 | ESI+ | 1.28503 | ↓ |
| 94 | O-dodecanoylcarnitine | Endogenous | 344.2790939 | 5.6677 | C02301 | ESI+ | 1.2803 | ↓ |
| 95 | Alpha-ketoisovaleric acid | Endogenous | 115.0399217 | 0.899786667 | C00141 | ESI- | 1.27492 | ↑ |
| 96 | Deoxyinosine | Endogenous | 251.078683 | 0.743493333 | C05512 | ESI- | 1.26904 | ↑ |
| 97 | Monoethyl malonic acid | Exogenous | 131.0350512 | 1.411325 | C00489 | ESI- | 1.26741 | ↓ |
| 98 | LysoPE(0:0/18:2(9Z,12Z)) | Exogenous | 476.277891 | 4.832983333 | C04438 | ESI- | 1.26566 | ↓ |
| 99 | Cucurbic acid | Endogenous | 211.1343533 | 3.738616667 | C16309 | ESI- | 1.26544 | ↓ |
| 100 | 2-Methyl-3-hydroxy-5-formylpyridine-4-carboxylate | Exogenous | 180.0305706 | 2.53655 | C06050 | ESI- | 1.2613 | ↑ |
| 101 | LysoPC(18:2(9Z,12Z)) | Exogenous | 520.3383182 | 4.87845 | C04230 | ESI+ | 1.25933 | ↓ |
| 102 | Linoleic acid | Endogenous | 281.2469404 | 6.73615 | C01595 | ESI+ | 1.25762 | ↓ |
| 103 | 3b,15b,17a-Trihydroxy-pregnenone | Endogenous | 349.2348909 | 5.75505 | C05475 | ESI+ | 1.25235 | ↓ |
| 104 | 4-Nitrophenol | Exogenous | 138.0195054 | 2.113683333 | C01020 | ESI- | 1.24943 | ↓ |
| 105 | Alpha-ketoisovaleric acid | Endogenous | 115.0399304 | 1.352519167 | C00141 | ESI- | 1.24172 | ↑ |
| 106 | Erythrodiol 3-palmitate | Endogenous | 375.275408 | 5.28445 | C17658 | ESI- | 1.23978 | ↓ |
| 107 | Linoleic acid | Endogenous | 281.2468534 | 7.463966667 | C01595 | ESI+ | 1.23964 | ↓ |
| 108 | Palmitoleoyl Ethanolamide | Endogenous | 298.2731693 | 5.8766 | C01595 | ESI+ | 1.2374 | ↓ |
| 109 | Octadecanedioic acid | Endogenous | 332.2793821 | 4.51095 | C14829 | ESI+ | 1.23155 | ↓ |
| 110 | PG(16:0/18:1(11Z)) | Endogenous | 747.519463 | 9.0384 | C00344 | ESI- | 1.2282 | ↑ |
| 111 | Palmitoylglycine | Endogenous | 314.2685753 | 5.704383333 | C14825 | ESI+ | 1.22524 | ↓ |
| 112 | Octyl gallate | Exogenous | 281.1395523 | 3.3232 | C15971 | ESI- | 1.19413 | ↑ |
| 113 | p-Hydroxyphenylacetic acid | Exogenous | 151.0400966 | 3.120158333 | C02519 | ESI- | 1.156 | ↓ |

Table 2. Differential metabolites identified between the MOD and QR groups.

(Table content would follow)
